# Supplementary figures and images for: Natural product-mediated reaction hijacking mechanism validates Plasmodium aspartyl-tRNA synthetase as an antimalarial drug target
Source: PLoS Pathog. 2025 Jul 8;21(7):e1013057. doi: 10.1371/journal.ppat.1013057 (PMC12262901; doi:10.1371/journal.ppat.1013057)

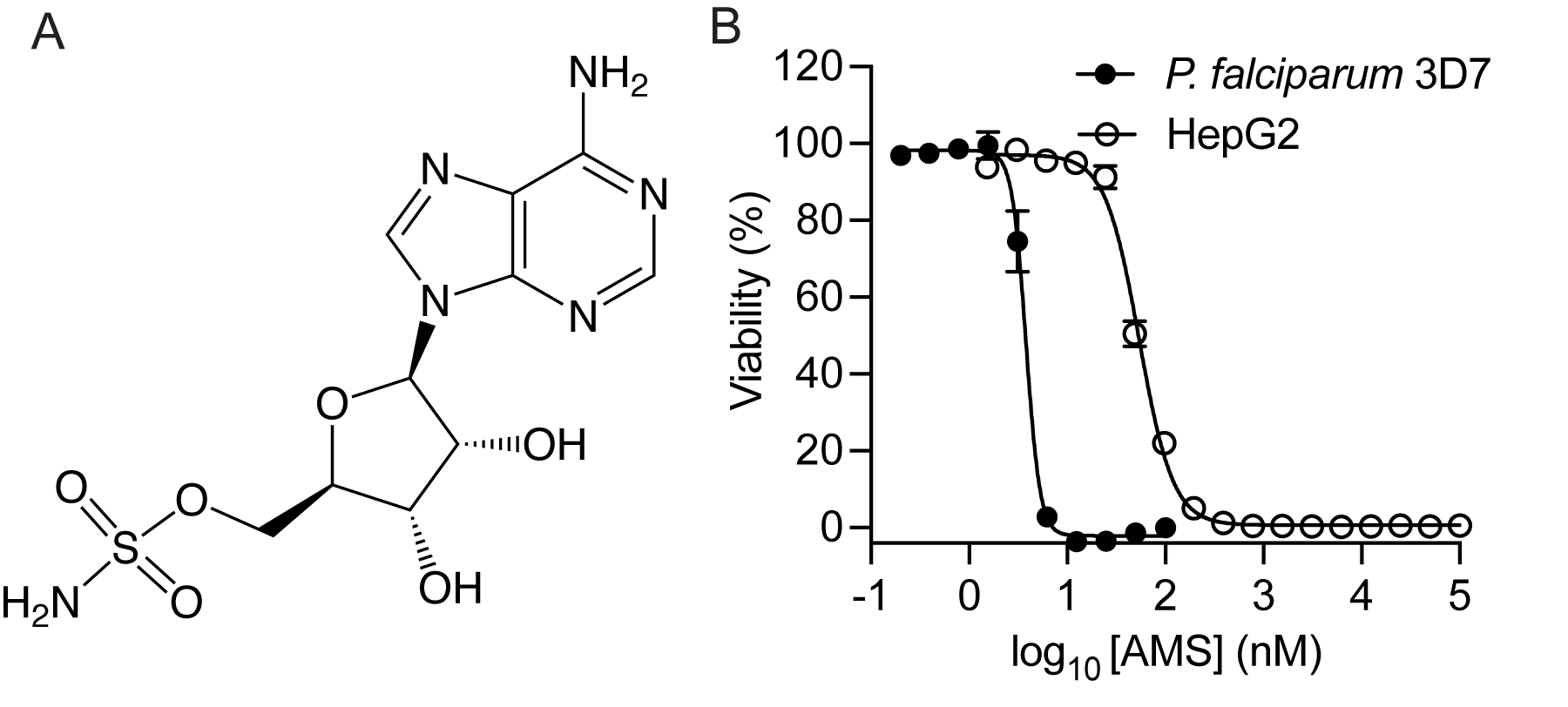

Supplement: S1 Fig — (A) Structure of AMS. (B) Sorbitol-synchronized ring stage parasites were subjected to a 72-h drug sensitivity assay with AMS (black circles). Data represent five independent experiments, each performed in duplicate. Cytotoxicity of AMS (white circles) against the HepG2 mammalian cell line in a 48-h exposure assay. Data represent five independent experiments, each performed in triplicate. Error bars indicate SEM. (TIF) [file ppat.1013057.s001.tif]

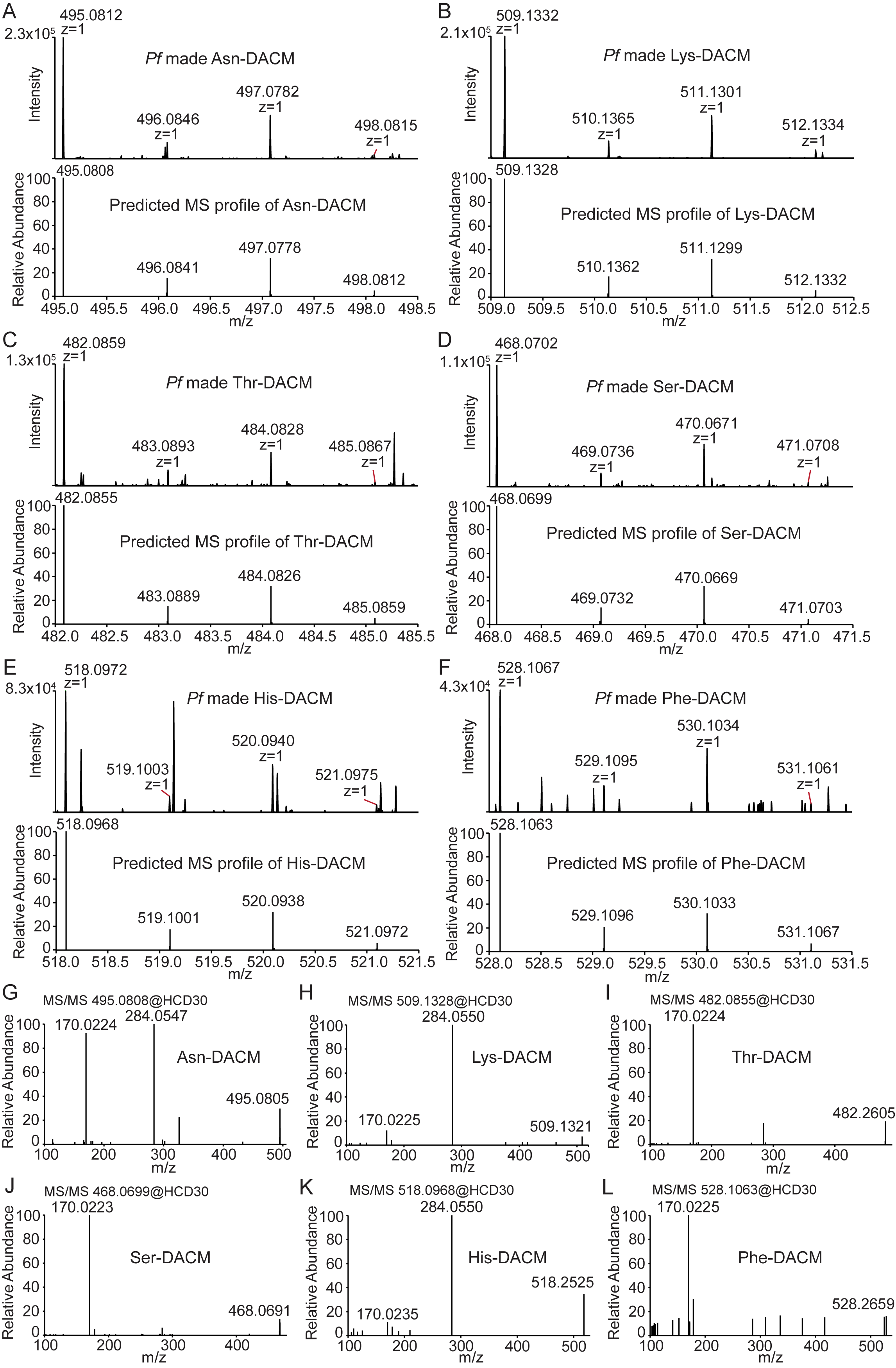

Supplement: S2 Fig — P. falciparum cultures were exposed to 10 µM DACM for 3 h. Parasite extracts were subjected to LC-MS/MS to search for DACM-amino acid conjugates. (A-F) Detected (top panels) and predicted mass spectra of (A) DACM-Asn (m/z = 495.0808), (B) DACM-Lys (m/z = 509.1328); (C) DACM-Thr (m/z = 482.0855); (D) DACM-Ser (m/z = 468.0699), (E) DACM-His (m/z = 518.0968); (F) DACM-Phe (m/z = 528.1063). (G-L). MS/MS spectra of the fragmented ions, including a m/z of 170.0228 found as a fragmented ion of each DACM-amino acid conjugate. (TIF) [file ppat.1013057.s002.tif]

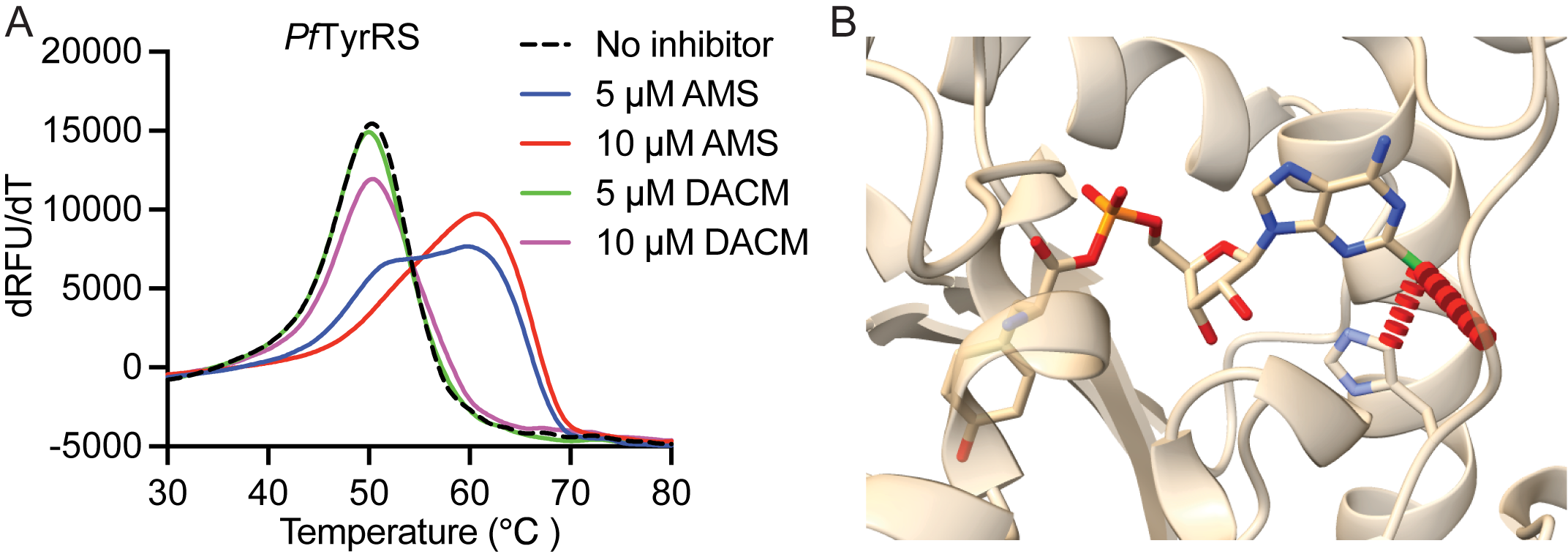

Supplement: S3 Fig — (A) First derivatives of melting curves of PfTyrRS (2.3 μM) after incubation at 37°C for 2 h with 10 μM ATP, 20 μM Tyr, 4 μM PftRNATyr, with 5 or 10 μM of AMS or DACM. Data are representative of three independent experiments. (B) A chlorine atom was added to the 2-position of the adenine ring system of Tyr-AMP at the active site of PfTyrRS (PDB: 7ROR) using ChimeraX [78]. Steric overlaps between the chlorine atom and the protein binding pocket are shown as red dashed rods. (TIF) [file ppat.1013057.s003.tif]

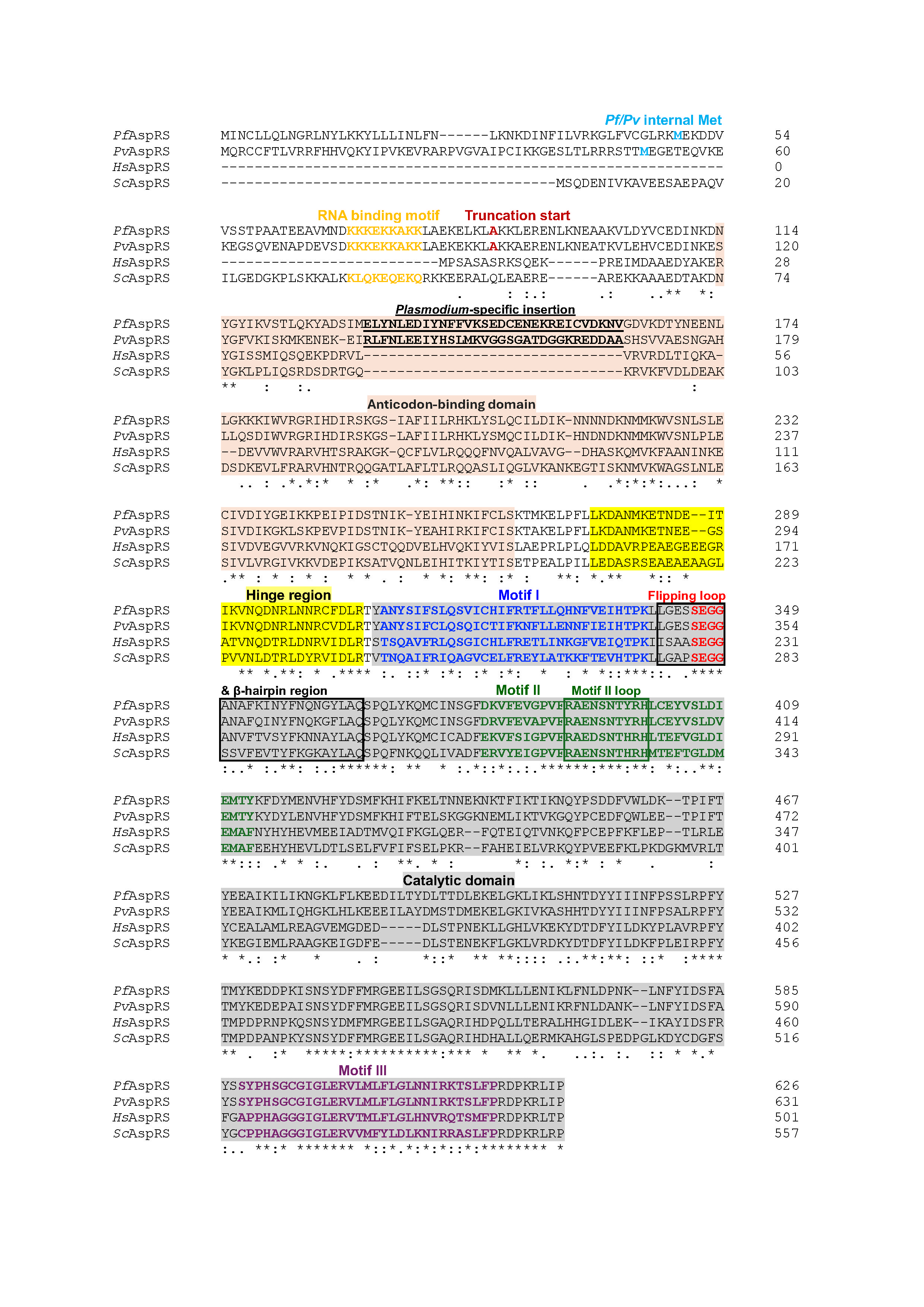

Supplement: S4 Fig — Alignment of AspRS sequences from P. falciparum (Pf), P. vivax (Pv), Homo sapiens (Hs), Saccharomyces cerevisiae (Sc), reveals a high level of conservation of the three Type II aaRS motifs (I-III, blue, green, purple text), which are involved in ATP binding and dimerization. The hinge region is highlighted in yellow. The Plasmodium sequences exhibit a large N-terminal extension with native initiation from an internal methionine (aqua text). The anticodon-binding domain (salmon) has a Plasmodium-specific insert (underlined). The flipping loop residues, SEGG, that have previously been shown to undergo dynamic motions that facilitate tRNA binding [40], are in red text. Two loops that are ordered in PvAspRS but disordered in HsAspRS are boxed, namely the flipping loop and flanking β-hairpin structure and the motif II loop. (TIF) [file ppat.1013057.s004.tif]

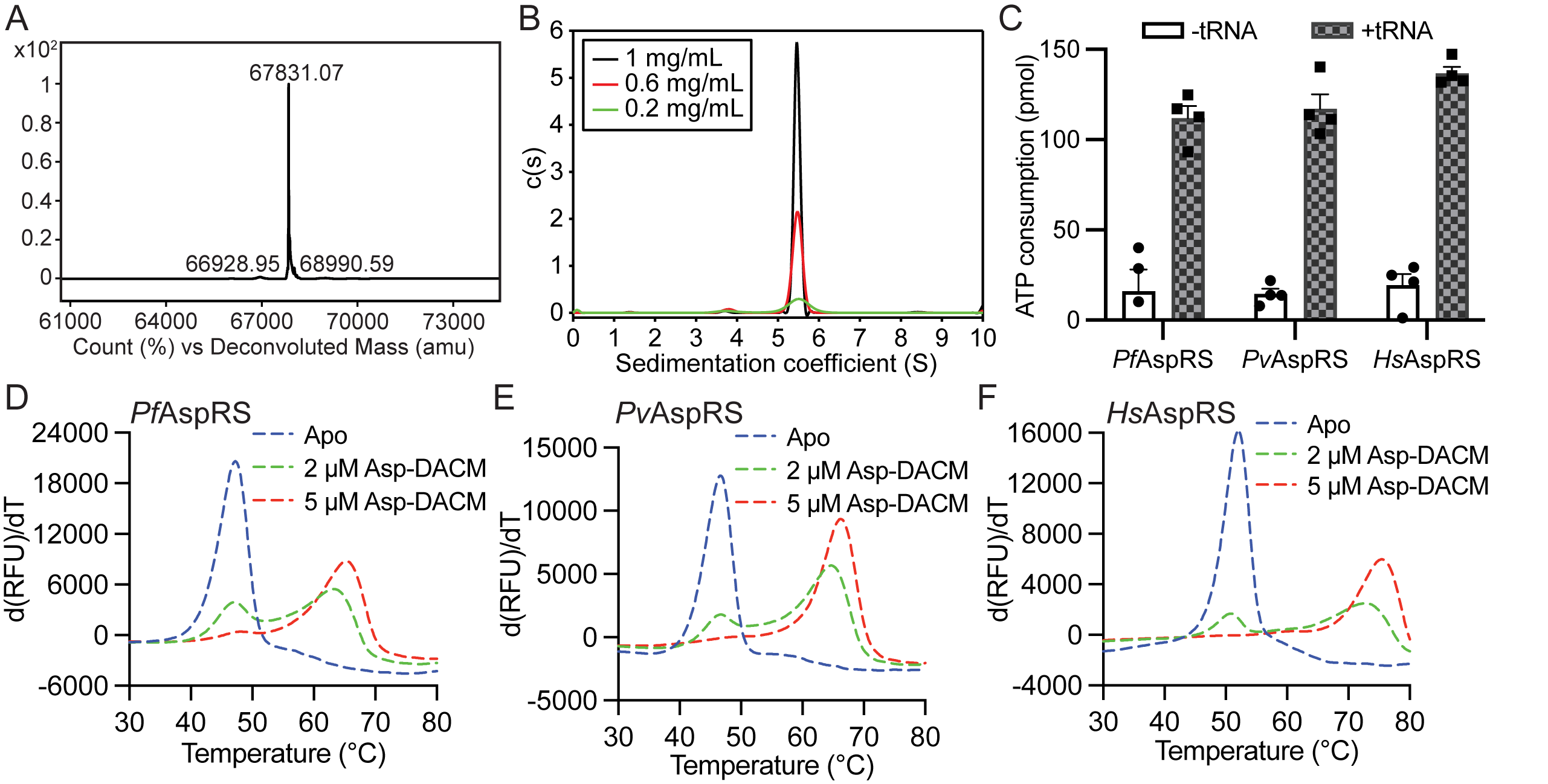

Supplement: S5 Fig — (A) Deconvoluted mass spectrum obtained using Agilent Mass Hunter software. The mass of the highest peak (67831 Da) correlates well with the theoretical mass of native length PfAspRS. (B) Sedimentation velocity analysis. Purified native length PfAspRS was diluted to 1, 0.6, and 0.2 mg/mL in buffer containing 50 mM Tris-HCl (pH 8), 150 mM NaCl, and 1 mM TCEP. Samples were subjected to analytical ultracentrifugation. Samples were centrifuged at 200,000 g and monitored at a wavelength of 290 nm. The continuous sedimentation coefficient c(s) was plotted as a function of the sedimentation coefficient (S). (C) ATP consumption by PfAspRS, PvAspRS and HsAspRS in the presence and absence of the EctRNA. Reagent concentrations: 50 nM PfAspRS and PvAspRS or 100 nM HsAspRS with 10 μM ATP, 200 μM Asp, 1 U/mL pyrophosphatase, 80 μM EctRNA for PfAspRS and PvAspRS and 160 μM EctRNA for HsAspRS. Data represent mean ± SEM from four independent experiments. (D-F) Thermal stabilization of native length AspRS enzymes by Asp-DACM. First derivatives of melting curves for native length PfAspRS (D), PvAspRS (E) or HsAspRS (F) (1.5 μM) after incubation at 37°C for 3 h with 2 or 5 μM Asp-DACM. Data are representative of three independent experiments. (TIF) [file ppat.1013057.s005.tif]

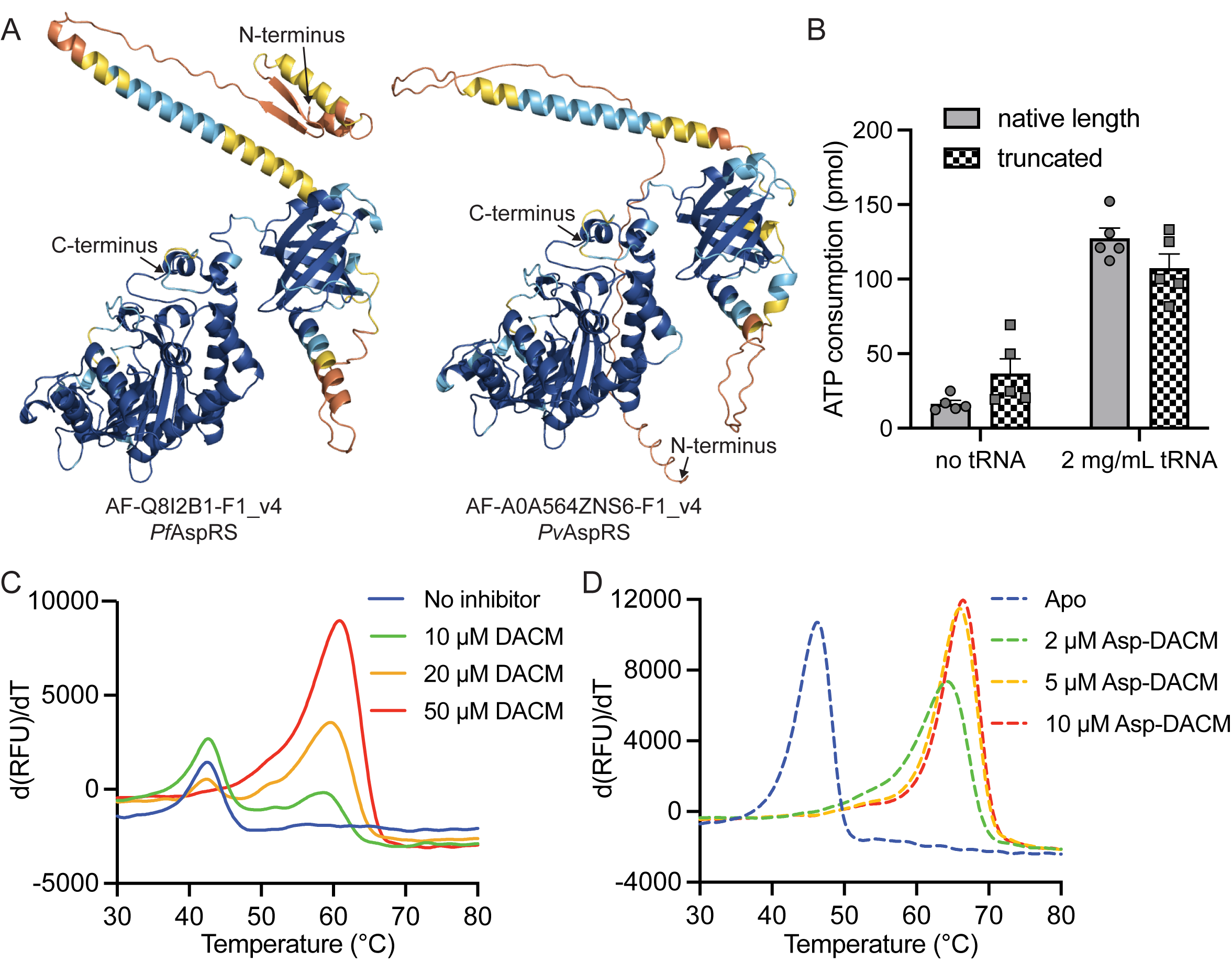

Supplement: S6 Fig — (A) AlphaFold predicted structures of full-length PfAspRS (PlasmoDB ID: PF3D7_0102900) and full-length PvAspRS (PlasmoDB ID: PVX_081610). Model confidence is predicted and colored. Blue: Very high (pLDDT > 90), sky blue (90 > pLDDT > 70), yellow (70 > pLDDT > 50), and orange: very low (pLDDT < 50) per-residue model confidence score (pLDDT). (B) ATP consumption by native length PvAspRS (51–631) and truncated PvAspRS (96–631) in the presence and absence of the EctRNA. 50 nM PvAspRS was incubated with 10 μM ATP, 200 μM Asp, 1 U/mL pyrophosphatase, ± 80 μM EctRNA in 25 mM Tris-HCl (pH 8), 150 mM NaCl, 5 mM MgCl2, 1 mM TCEP, 0.1 mg/mL BSA for 1 h at 37°C. Data represent five independent experiments. (C, D) First derivatives of melting curves for truncated PvAspRS (1.5 μM) in apo form or after incubation at 37°C for 3 h with 10 μM ATP, 20 μM Asp, 80 μM EctRNA, 10–50 μM DACM (C) or 2–10 μM Asp-DACM (D). Data are representative of three independent experiments. (TIF) [file ppat.1013057.s006.tif]

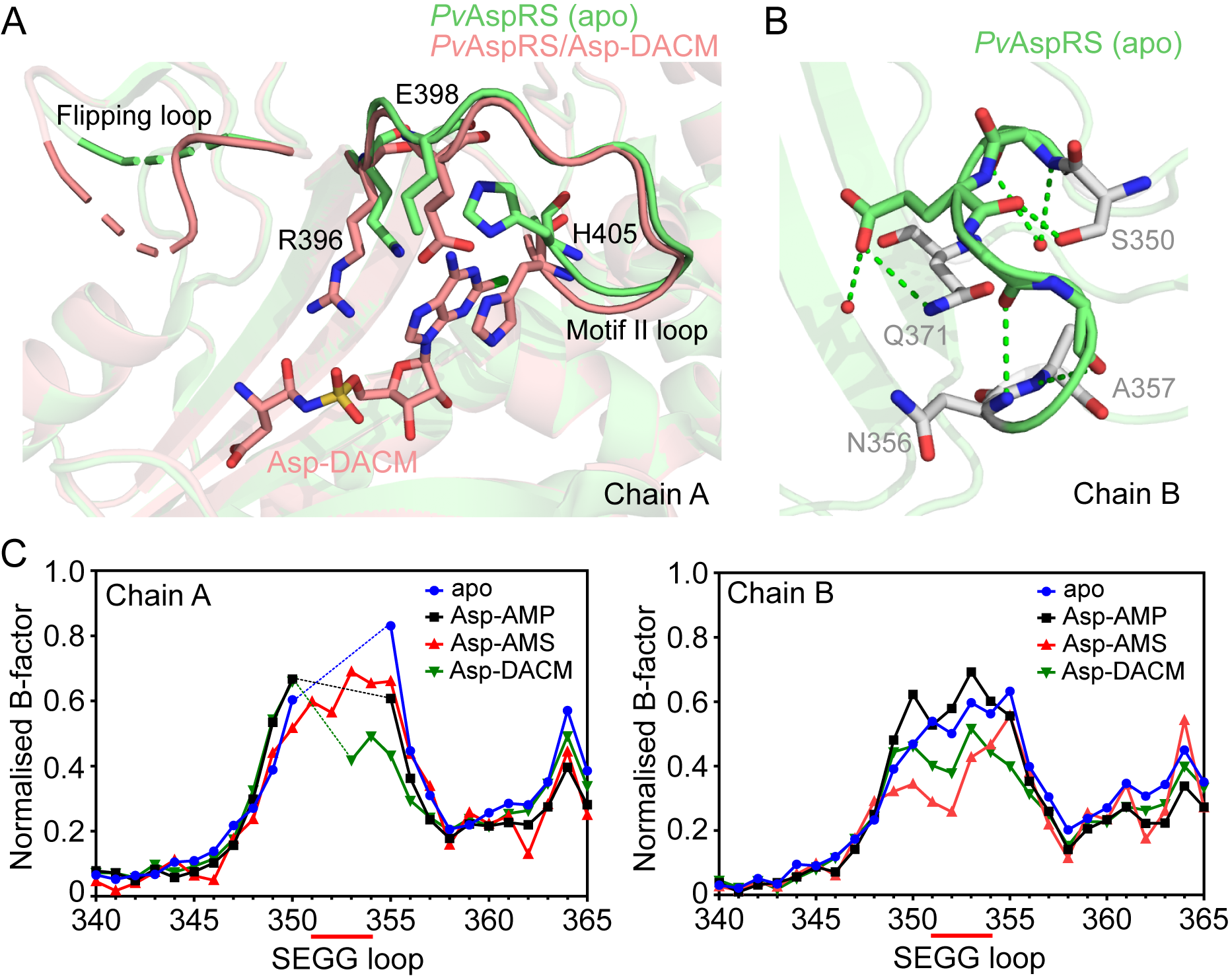

Supplement: S7 Fig — (A) Overlay of chain A of apo PvAspRS and Asp-DACM-bound PvAspRS showing the flipping loop and the motif II loop. (B) Ribbon representation of the flipping loop of chain B of apo PvAspRS. The SEGG loop interacts with a conserved residue Q371, as well as N356, S350 and A357. (C) B-factor analysis of the chain A and B flipping loops of different PvAspRS structures. The x-axis shows residue number. (TIF) [file ppat.1013057.s007.tif]

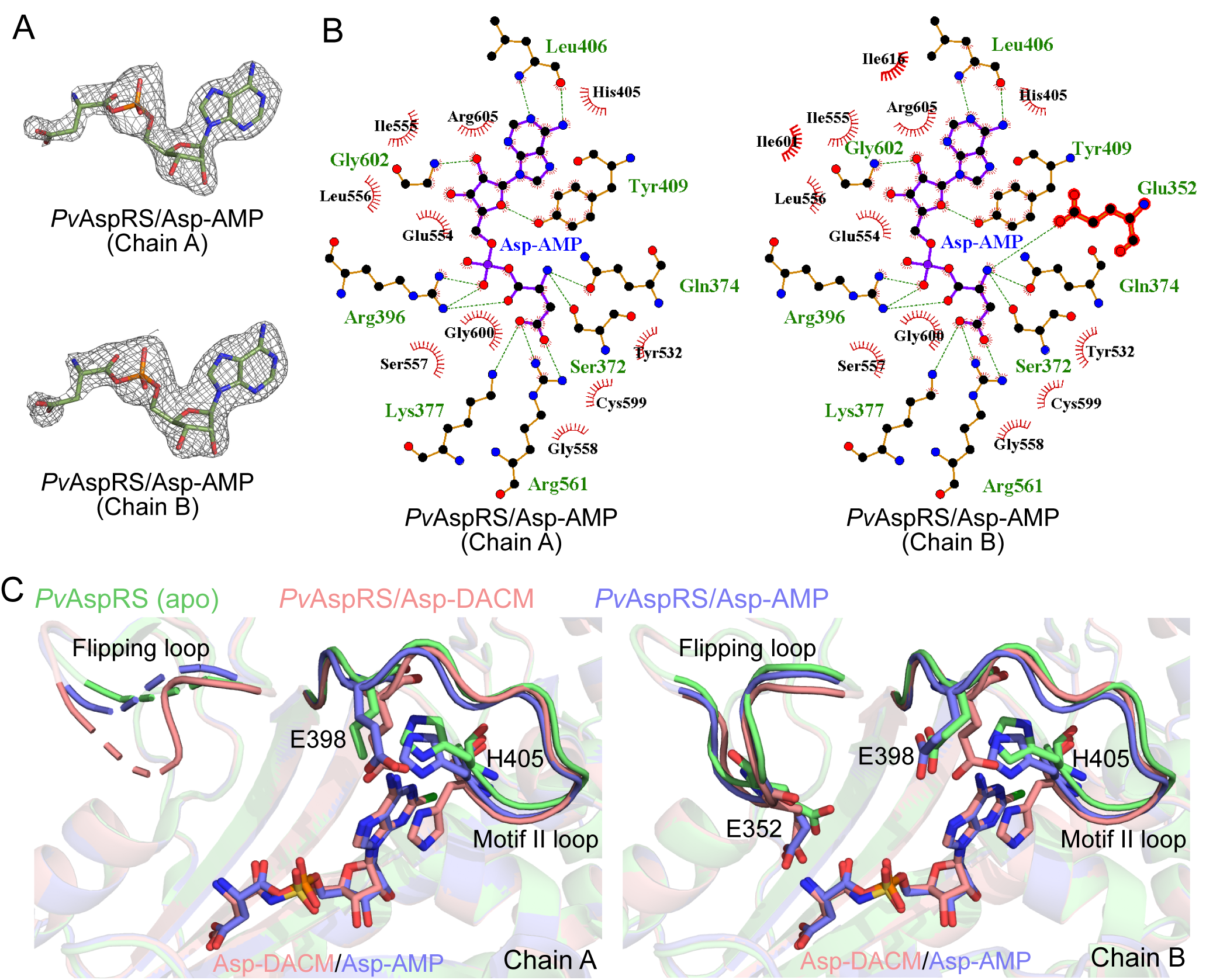

Supplement: S8 Fig — (A) 2Fo-Fc maps contoured at 2 σ (mesh surface) showing electron density supporting the position of Asp-AMP bound to chains A and B. (B) Ligplots of Asp-AMP active site interfaces for the A and B chains. Hydrogen bonds and salt bridges are depicted with dashed (green) lines. Other interactions between protein and ligand are indicated by red arcs. (C) Overlay of PvAspRS (Apo), PvAspRS (Asp-DACM) and PvAspRS (Asp-AMP) showing the flipping loop and the motif II loop. Chain A (left), Chain B (right). The sidechain of residue E398 of apo PvAspRS Chain A is not built due to insufficient density. (TIF) [file ppat.1013057.s008.tif]
