# Supplementary material for: Natural product-mediated reaction hijacking mechanism validates Plasmodium aspartyl-tRNA synthetase as an antimalarial drug target
Source: PLoS Pathog. 2025 Jul 8;21(7):e1013057. doi: 10.1371/journal.ppat.1013057 (PMC12262901; doi:10.1371/journal.ppat.1013057)
Supplement: S1 Table — *N.D. = Not determined due to incomplete transition. (PDF) [file ppat.1013057.s009.pdf]

S1 Table. Thermal stabilization of recombinant *Pf*TyrRS, native length *Pf, Pv, Hs*AspRS and truncated *Pv*AspRS by nucleoside sulfamates. *N.D. = Not determined due to incomplete transition.

		Tm_{app} (°C) Mean ± SEM	Δ Tm_{app} (°C)	n
***Pf*TyrRS**	*Pf*TyrRS alone	49.3 ± 0.0	-	3
	*Pf*TyrRS + ATP + Asp + *Pf*tRNA(Tyr)	50.4 ± 0.1	-	3
	*Pf*TyrRS + ATP + Asp + *Pf*tRNA(Tyr) + 5 µM DACM	50.0 ± 0.1	-0.3	3
	*Pf*TyrRS + ATP + Asp + *Pf*tRNA(Tyr) + 10 µM DACM	50.8 ± 0.6	0.4	3
	*Pf*TyrRS + ATP + Asp + *Pf*tRNA(Tyr) + 5 µM AMS	N.D.*	N.D.*	3
	*Pf*TyrRS + ATP + Asp + t *Pf*tRNA(Tyr) + 10 µM AMS	60.6 ± 0.4	10.2	3
***Pf*AspRS** (49-626)	*Pf*AspRS alone	47.3 ± 0.2	-	3
	*Pf*AspRS + ATP + Asp + *Ec*tRNA	43.0 ± 0.5	-	6
	*Pf*AspRS + ATP + Asp + *Ec*tRNA + 10 µM DACM	N.D.*	N.D.*	3
	*Pf*AspRS + ATP + Asp + *Ec*tRNA + 20 µM DACM	N.D.*	N.D.*	3
	*Pf*AspRS + ATP + Asp + *Ec*tRNA + 50 µM DACM	61.1 ± 0.1	18.1	3
	*Pf*AspRS + 2 µM Asp-DACM	N.D.*	N.D.*	3
	*Pf*AspRS + 5 µM Asp-DACM	65.3 ± 0.1	18.0	3
	*Pf*AspRS + 10 µM Asp-DACM	65.7 ± 0.1	18.4	3
	*Pf*AspRS + ATP + Asp + *Ec*tRNA + 10 µM AMS	N.D.*	N.D.*	3
	*Pf*AspRS + ATP + Asp + *Ec*tRNA + 20 µM AMS	56.4 ± 0.2	13.4	3
	*Pf*AspRS + ATP + Asp + *Ec*tRNA + 50 µM AMS	57.0 ± 0.2	14.0	3
***Pv*AspRS** (51-631)	*Pv*AspRS alone	46.8 ± 0.03	-	3
	*Pv*AspRS + ATP + Asp + *Ec*tRNA	43.4 ± 0.3	-	6
	*Pv*AspRS + ATP + Asp + *Ec*tRNA + 10 µM DACM	62.4 ± 0.02	19.0	3
	*Pv*AspRS + ATP + Asp + *Ec*tRNA + 20 µM DACM	62.6 ± 0.2	19.2	3
	*Pv*AspRS + ATP + Asp + *Ec*tRNA + 50 µM DACM	62.6 ± 0.2	19.2	3
	*Pv*AspRS + 2 µM Asp-DACM	N.D.*	N.D.*	3
	*Pv*AspRS + 5 µM Asp-DACM	66.3 ± 0.005	19.5	3
	*Pv*AspRS + 10 µM Asp-DACM	66.8 ± 0.05	20.0	3
	*Pv*AspRS + ATP + Asp + *Ec*tRNA + 10 µM AMS	59.3 ± 0.3	15.9	3
	*Pv*AspRS + ATP + Asp + *Ec*tRNA + 20 µM AMS	59.3 ± 0.2	15.9	3

	*Pv*AspRS + ATP + Asp + *Ec*tRNA + 50 μM AMS	59.5 ± 0.2	16.1	3
***Hs*AspRS** (1-501)	*Hs*AspRS alone	52.1 ± 0.02	-	3
	*Hs*AspRS + ATP + Asp + *Ec*tRNA	49.1 ± 0.2	-	6
	*Hs*AspRS + ATP + Asp *Ec*tRNA + 10 μM DACM	N.D.*	N.D.*	3
	*Hs*AspRS + ATP + Asp + *Ec*tRNA + 20 μM DACM	N.D.*	N.D.*	3
	*Hs*AspRS + ATP + Asp + *Ec*tRNA + 50 μM DACM	N.D.*	N.D.*	3
	*Hs*AspRS + 2 μM Asp-DACM	N.D.*	N.D.*	3
	*Hs*AspRS + 5 μM Asp-DACM	75.5 ± 0.1	23.4	3
	*Hs*AspRS + 10 μM Asp-DACM	76.0 ± 0.2	23.9	3
	*Hs*AspRS + ATP + Asp + *Ec*tRNA + 10 μM AMS	48.7 ± 0.3	-0.4	3
	*Hs*AspRS + ATP + Asp + *Ec*tRNA + 20 μM AMS	48.6 ± 0.1	-0.5	3
	*Hs*AspRS + ATP + Asp + *Ec*tRNA + 50 μM AMS	48.7 ± 0.2	-0.4	3
***Pv*AspRS** (96-631)	*Pv*AspRS (96-631) alone	46.3 ± 0.2	-	3
	*Pv*AspRS (96-631) + ATP + Asp + *Ec*tRNA	42.5 ± 0.2	-	3
	*Pv*AspRS (96-631)+ ATP + Asp + *Ec*tRNA + 10 μM DACM	N.D.*	N.D.*	3
	*Pv*AspRS (96-631)+ ATP + Asp + *Ec*tRNA + 20 μM DACM	N.D.*	N.D.*	3
	*Pv*AspRS (96-631)+ ATP + Asp + *Ec*tRNA + 50 μM DACM	60.9 ± 0.1	18.4	3
	*Pv*AspRS + 2 μM Asp-DACM	64.2 ± 0.1	17.9	3
	*Pv*AspRS + 5 μM Asp-DACM	66.0 ± 0.2	19.7	3
	*Pv*AspRS + 10 μM Asp-DACM	66.4 ± 0.0	20.1	3
