# Supplementary material for: Natural product-mediated reaction hijacking mechanism validates Plasmodium aspartyl-tRNA synthetase as an antimalarial drug target
Source: PLoS Pathog. 2025 Jul 8;21(7):e1013057. doi: 10.1371/journal.ppat.1013057 (PMC12262901; doi:10.1371/journal.ppat.1013057)
Supplement: S2 Table — (PDF) [file ppat.1013057.s010.pdf]

S2 Table. Protein–ligand docking scores determined using the Surflex fragment matching strategy.

Compound	Tyrosine fragment docking score
Tyr-AMP	15.5
Tyr-AMS	15.0
Tyr-DACM	13.6
