# Supplementary material for: Natural product-mediated reaction hijacking mechanism validates Plasmodium aspartyl-tRNA synthetase as an antimalarial drug target
Source: PLoS Pathog. 2025 Jul 8;21(7):e1013057. doi: 10.1371/journal.ppat.1013057 (PMC12262901; doi:10.1371/journal.ppat.1013057)
Supplement: S3 Table — (PDF) [file ppat.1013057.s011.pdf]

S3 Table. X-ray diffraction data collection and refinement statistics for truncated apo *Pv*AspRS and in complex with ligands Asp-DACM, Asp-AMP and Asp-AMS.
Values for the highest resolution shell are given in parentheses.

† *R*sym = ΣhklΣi |Ii (hkl) - 〈I(hkl)〉 | / ΣhklΣiIi (hkl)

§ *R*meas= Σhkl [N/(N - 1)]$^{1/2}$ Σi |Ii (hkl) - 〈I (hkl)〉 | / Σhkl Σi Ii (hkl)

‡ *R*pim= Σhkl [1/(N - 1)]$^{1/2}$ Σi |Ii (hkl) - 〈I (hkl)〉 | / Σhkl Σi Ii (hkl)

$CC_{1/2}$ = Pearson correlation coefficient between independently merged half data sets.

**Molecule**	***Pv*AspRS_Apo**	***Pv*AspRS (Asp-DACM)**	***Pv*AspRS (Asp-AMP)**	***Pv*AspRS (Asp-AMS)**
**Data collection**				
Space group	$P\ 6_1\ 2\ 2$	$P\ 6_1\ 2\ 2$	$P\ 6_1\ 2\ 2$	$P\ 6_1\ 2\ 2$
Wavelength	0.97625	0.95373	0.97625	0.97625
Number of images	3600	3600	3600	3600
Oscillation range per image (°)	0.1	0.1	0.1	0.1
Detector	Eiger2 XE 16M	Eiger 16M	Eiger2 XE 16M	Eiger2 XE 16M
Cell dimensions				
a, b, c (Å)	140.447, 140.447, 272.559	140.045, 140.045, 273.02	139.939, 139.939, 271.801	140.253, 140.253, 267.611
α, β, γ (°)	90, 90, 120	90, 90, 120	90, 90, 120	90, 90, 120
Resolution (Å)	90.85 - 2.06 (2.10 - 2.06)	48.88 - 2.36 (2.38 - 2.36)	121.19 – 2.14 (2.18 - 2.14)	121.46 - 1.84 (1.87-1.84)
*R*sym†	0.109 (2.017)	0.1154 (1.424)	0.118 (4.326)	0.220 (5.537)
*R*meas§	0.110 (2.732)	0.1169 (1.443)	0.120 (4.469)	0.223 (5.653)
*R*pim‡	0.018 (0.750)	0.01836 (0.2264)	0.020 (1.100)	0.035 (1.132)
$CC_{1/2}$	1.000 (0.323)	0.999 (0.839)	1.000 (0.352)	0.999 (0.366)
Mean $I/\sigma(I)$	20.4 (0.3)	24.15 (2.58)	23.4 (0.6)	14.7 (0.7)
Total observations	3254813 (55961)	2661445 (168870)	2806409 (67161)	5281504 (165007)
Unique reflections	98198 (4723)	65749 (1952)	80749 (4253)	134470 (6630)
Completeness (%)	99.8 (97.6)	99.70 (90.96)	92.6 (99.0)	100 (100)
Multiplicity	33.1 (11.8)	40.5 (38.4)	34.8 (15.8)	39.3 (24.9)
Wilson *B* factor (Å^2)	47.00	50.43	51.58	29.30
**Refinement**				
Resolution (Å)	72.83 – 2.06 (2.08 – 2.06)	48.88 - 2.356 (2.38 - 2.36)	60.59 – 2.14 (2.16 – 2.14)	50.20 - 1.84 (1.86 - 1.84)
Reflections used in refinement	97555 (2630)	65749 (4445)	80475 (2690)	134353 (4125)
R_{free} reflections	4843 (144)	2000 (135)	4010 (146)	6602 (206)
R_{work}	0.1830 (0.3769)	0.1876 (0.2802)	0.1838 (0.3538)	0.1677 (0.3443)
R_{free}	0.2111 (0.3782)	0.2146 (0.3055)	0.2215 (0.3599)	0.1956 (0.3665)
Protein molecules in ASU	2	2	2	2

Total non-hydrogen atoms	8598	8381	8665	9167
Protein	8061	7973	8066	8233
Ligand/ion	28	76	82	86
Solvent	509	332	517	930
Mean B-factor (Å^2)	58.1	58.2	63.1	36.8
Protein	58.2	58.5	63.3	36.1
Ligand/ion	74.7	49.9	72.0	29.0
Solvent	56.1	53.1	60.2	44.55
RMS deviations				
Bond lengths (Å) (outliers > 4σ)	0.007	0.007	0.007	0.008
Bond angles (°) (outliers > 4σ)	0.847	0.820	0.893	0.961
Rotamer outliers (%)	0.92	1.17	0.70	0.11
Clashscore	3.21	1.61	4.50	3.14
Cβ outliers	0	0	0	0
Molprobity score	1.30	1.62	1.44	1.35
Ramachandran Plot				
Favored (%)	97.58	95.73	97.39	97.67
Allowed (%)	2.42	4.27	2.62	2.33
Outliers (%)	0.00	0.00	0.00	0.00
PDB code	9M5M	9NPJ	9M5N	9M5O
