# Supplementary material for: Natural product-mediated reaction hijacking mechanism validates Plasmodium aspartyl-tRNA synthetase as an antimalarial drug target
Source: PLoS Pathog. 2025 Jul 8;21(7):e1013057. doi: 10.1371/journal.ppat.1013057 (PMC12262901; doi:10.1371/journal.ppat.1013057)
Supplement: S1 Text — (DOCX) [file ppat.1013057.s012.docx]

**Chemistry Methods**

**General Chemistry**

All reactions were carried out in dry solvents under anhydrous conditions unless otherwise stated. All chemicals were purchased from commercial suppliers and used without further purification. 2-Chloro-2ʹ,3ʹ-*O-*isopropylidene-5ʹ-*O-*sulfamoyl adenosine (**2**) and 2-chloro-5ʹ-*O-*sulfamoyl adenosine (**DACM**) were prepared according to literature methods [1] [2]. All reactions were monitored by TLC using silica plates with visualisation of eluted bands by UV fluorescence (λ = 254 nm) and charring with vanillin stain (6 g vanillin in 100 mL of EtOH containing 1% v/v 98% Sulfuric acid. Silica gel flash chromatography was performed using silica gel 60 Å (230-400 mesh). NMR (^1^H, ^13^C, COSY, NOESY, HSQC and HMBC) spectra were recorded on either a Bruker AVANCE III HD 400/500 MHz NMR spectrometer equipped with a BBO probe at 25 °C or a Bruker AVANCE III HD 800 NMR equipped with Triple (TCI) Resonance 5 mm Cryoprobe. Chemical Shifts for ^1^H and ^13^C NMR obtained in DMSO-*d*_6_ are reported in ppm relative to residual solvent proton (δ = 2.50 ppm) and carbon (δ = 39.5 ppm) signals, respectively. Chemical Shifts for ^1^H and ^13^C NMR obtained in methanol-d_4_ are reported in ppm relative to residual solvent proton (δ = 3.31 ppm) and carbon (δ = 49.0 ppm), signals, respectively. Chemical Shifts for ^1^H and ^13^C NMR obtained in CDCl_3_ are reported in ppm relative to residual solvent proton (δ= 7.26 ppm), and carbon (δ= 77.2 ppm) signals, respectively. Signal splitting multiplicity is indicated as follows: s (singlet), d (doublet), t (triplet), q (quartet), m (multiplet), dd (doublet of doublets), br (broad signal), a (apparent). Assignments of ^1^H and ^13^C chemical shifts were established by COSY, HSQC, HMBC, and NOESY experiments. Coupling constants are reported in hertz (Hz). LRMS (ESI) data were acquired on a Thermo Fisher MSQ Plus single quadruple ESI mass spectrometers using electrospray as the ionisation technique in positive and/or negative mode as stated. HRMS (ESI) data were acquired on a Bruker MaXis II QTOF mass spectrometer using an ESI source the ionisation technique in positive-ion and/or negative mode as stated. All MS analysis samples were prepared as solutions in either methanol or acetonitrile. Purity of the compounds were >95% as determined by Thermo Fisher Dionex Ultimate 3000 series HPLC via UV detection at 254 nm. The purity of the **Asp-AMS** product was confirmed by analytical HPLC (Shimadzu Prominence semi-preparative LC-20AR) using a Sim-pack GIST C-18 column (5 µM, 250 mm length, 4.6 mm I.D.) at a flow rate of 1 mL/min.

**Synthesis of Asp-DACM**

**Scheme 1**. Synthesis of Asp-DACM via coupling of Asp(Boc)-*tert*-butyl ester *N*-hydroxy succinate (**1**) to the 2-chloro-2ʹ,3ʹ-*O*-isopropylidene-5ʹ-*O*-sulfamoyl adenosine (**2)** followed by global deprotection mediated by trifluoroacetic acid (TFA).

**Synthesis of Asp-AMS**

|  |
| --- |
| **Scheme 2.** The synthetic strategy for the preparation of **Asp-AMS.** Reagents and conditions. (i) TBSCl, DMAP, imidazole, DMF, rt 12 h; (ii) TFA: H_2_O (1:1), THF, 45 min; (iii) chlorosulfonyl isocyanate, HCOOH, acetonitrile, DMA, rt, 2 h; (iv) DIPEA, DMF, 24 h, room temperature (rt); piperidine, THF, 45min; (v) TBAF, THF 0 ℃ to rt 5 h; (iv) 4N HCl-Dioxane, THF, 2 h. |

**l-Aspartyl (Boc)-*tert*-butyl ester *N*-hydroxysuccinate (1)**

Asp(Boc)-*tert*-butyl ester (0.2000 g, 0.6913 mmol), *N*-hydroxy succinimide (0.0955 g, 0.8296 mmol, 1.2 equiv) and *N*,*N*'-dicyclohexylcarbodiimide (DCC) (0.1458 g, 0.7604 mmol, 1.1 equiv) were dissolved in THF (3.5 mL) and stirred at rt for 1.5 h after which TLC indicated complete consumption of the starting material. The mixture was filtered, the precipitate washed with THF (10 mL) and the solvent was removed under reduced pressure. The title compound **1** (0.2442 g, 0.6319 mmol, 91%) was obtained as a colourless solid and carried forward as crude without purification and characterisation.

**2-Chloro-2',3'-*O*-isopropylidene-5'-*O*-[*N*-(Boc-l-aspartyl)sulfamoyl]adenosine *tert*-butyl ester (3)**

To a solution of 2-chloro-2ʹ,3ʹ-*O-*isopropylidene-5ʹ-*O-*sulfamoyl adenosine (0.0500 g, 0.1188 mmol) (**2**) and **1** (0.0918 g, 0.2376 mmol, 2.0 equiv) in DMF (1.0 mL), 1,8-diazobicyclo[5.4.0]undec-7-ene (DBU) (0.0181 g, 0.0177 mL, 0.118 mmol. 1.0 eq.) was added and the mixture was stirred at rt for 18 h. The crude product was precipitated from diethyl ether (50 mL) and then dissolved in MeOH (25 mL). To this solution, aminomethylated resin (pre-washed with MeOH and dried) was added and stirred at rt for 1.5 h. The mixture was filtered, and the resin washed with MeOH (15 mL). The solvent was removed under reduced pressure and the crude product was purified by silica gel flash chromatography (5% 🡪 10% MeOH in CH_2_Cl_2_) to afford the title compound 3 as a white solid (0.0157 g, 0.0227 mmol, 19%). *R_f_* 0.53 (10% MeOH in CH_2_Cl_2_). ^1^H NMR (800 MHz, Methanol-*d*_4_): δ 8.36 (s, 1H, H-8), 6.17 (d, J = 2.9 Hz, 1H, H-1ʹ), 5.32 (dd, J = 6.1, 3.0 Hz, 1H, H-2ʹ), 5.08 (dd, J = 6.1, 2.5 Hz, 1H, H-3ʹ), 4.53 (td, J = 4.1, 2.4 Hz, 1H, H-4ʹ), 4.34 – 4.30 (m, 1H, H-Asp_(α)_), 4.27 (d, J = 4.2 Hz, 2H, H-5ʹ and H-5ʹʹ), 2.77 (dd, J = 15.6, 4.9 Hz, 1H, H-Asp_(β)_), 2.62 (dd, J = 15.6, 7.6 Hz, 1H, H-Asp_(β)_), 1.61 (s, 3H, iPr-CH_3_), 1.42 (d, J = 2.8 Hz, 18H, 2 × ^t^Bu-CH_3_), 1.39 (s, 3H, iPr-CH_3_).^13^C{H} NMR (200 MHz, Methanol-*d*_4_): δ 179.9 (Asp-*C*ONH-), 172.2 (Asp-γ-*C*O_2_-*^t^*Bu), 158.1 (C-6), 157.7 (BOC-*C*O-N), 155.5 (C-2), 151.7 (C-4), 141.6 (C-8), 119.1 (C-5), 115.4 (iPr-*C*H), 91.7 (C-1ʹ), 85.74 (C-4ʹ), 85.67 (C-2ʹ), 83.1 (C-3ʹ), 82.1 (^t^Bu-(*C*CH_3_)_3_), 80.5 (^t^Bu-(*C*CH_3_)_3_), 70.0 (C-5’), 55.0 (Asp-α-CH), 39.9 (Asp-β-CH), 28.8 (3 × ^t^Bu-(C*C*H_3_)_3_), 28.4 (3 × ^t^Bu-(C*C*H_3_)_3_), 27.5 (iPr-*C*H_3_), 25.6 (iPr-*C*H_3_). *m/z* (LRMS ESI) 692.2 [M-H]^+^, 690.2 [M-H]^-^. *m/z* (HRMS ESI^+^) [M+H]^+^ found 692.2111: calcd for C_26_H_39_ClN_7_O_11_S 692.2117.

**2-Chloro-5ʹ-*O*-[*N*-(l-aspartyl)-sulfamoyl] adenosine (Asp-DACM)**

Compound **3** (0.0110 g, 0.0159 mmol) was dissolved in methanol (100 µL, 2.64 mmol), then trifluoro acetic acid (25.0 µL, 0.326 mmol, 20.5 equiv) and water (25.7 µL, 1.43 mmol, 90 equiv) added. The mixture was placed on a rotary evaporator and put under a mild vacuum (~500 mbar) for 2 h. Upon complete consumption of the starting material as determined by TLC the solution was evaporated to dryness *in vacuo.* The crude compound was resuspended in acetonitrile (~0.5 mL) and triethylamine (20 µL) added then centrifuged and the supernatant removed. The pellet was resolubilised in MeOH (~1 mL) transferred to storage vial and concentrated *in vacuo* to yield a transparent film of sufficiently pure compound (0.0069 g, 0.0139 mmol, 88%). ^1^H NMR (500 MHz, DMSO-d_6_): δ = 8.39 (s, 1H, H-8), 8.10 – 7.73 (m, 5H, NH, Asp-NH_2_, NH_2_-6), 5.84 (d, *J* = 5.9 Hz, 1H H-1ʹ), 4.51 (dd, *J* = 5.9, 4.6 Hz, 1H, H-2ʹ), 4.22 – 4.07 (m, 4H, H-3ʹ, H-4ʹ, H-5ʹ_(α and β)_), 3.77 (d, *J* = 7.8 Hz, 1H, H-Asp_(α)_), 2.86 (dd, *J* = 17.4, 4.3 Hz, 1H, H-Asp_(βʹ)_), 2.64 (dd, *J* = 17.4, 7.7 Hz, 1H, H-Asp_(β″ )_).^13^C{H} NMR (125 MHz, DMSO-d_6_): δ = 171.6 (Asp-*C*O_2_H), 170.8 (Asp-*C*ONH-), 156.8 (C-6), 153.2 (C-2), 150.6 (C-4), 139.6 (C-8), 117.9 (C-5), 86.9 (C-1ʹ), 82.6 (C-3ʹ), 73.7 (C-2ʹ), 70.6 (C-4ʹ), 67.9 (C-5ʹ), 51.2 (C- Asp_(α)_), 35.4 (Asp_(β)_). *m/z* (LRMS ESI^-^) 494.1 [M-H]^-^. *m/z* (HRMS ESI^-^) [M-H]^-^ found 494.0514: calcd for C_14_H_17_ClN_7_O_9_S 494.0502.

**2′,3′,5′-Tris-O-((1,1-dimethylethyl) dimethyl silyl) adenosine (5)** [3]

Adenosine **4** (1 g, 3.74 mmol), DMAP (45.6 mg, 0.37 mmol), and imidazole (2.3 g, 33.61 mmol) were dissolved in dry DMF (15 mL). After stirring for 5 min, TBS-Cl (2.8 g, 18.70 mmol) was added portion-wise, and the reaction stirred at room temperature for 24 h. After completion of the reaction, the mixture was quenched with aq. NaHCO_3_ (30 mL). The resulting mixture was extracted with ethyl acetate (3 × 30 mL), and the combined organic extract was dried over Na_2_SO_4_. The solvent was evaporated under reduced pressure and the crude product was purified by silica gel column chromatography (40% ethyl acetate in pet ether), to obtain compound **5** as a white solid (2.2 g, 96%); R*_f_* = 0.3 (40% ethyl acetate in pet ether ); mp 136-138 ℃; ^1^H NMR (400 MHz, CDCl_3_) δ: 8.34 (s, 1H), 8.17 (s, 1H), 6.19 (s, 2H), 6. 02 (d, 5.0 Hz, 1H), 4.69 (t, *J* = 4.6 Hz, 1H), 4.33 (t, *J* = 4.8 Hz, 1H), 4.12 (q, *J* = 4.5 Hz, 1H) 4.02 (dd, *J* = 11.5, 4.5, Hz, 1H), 3.78 (dd, *J* = 11.5, 3.1 Hz, 1H), 0.96 (s, 9H), 0.94 (s, 9H), 0.80 (s, 9H), 0.14-0.11 (m, 12H), −0.02 (s, 3H), −0.20 (s, 3H); ^13^C NMR (100 MHz, CDCl_3_) δ: 155.3,152.9, 149.9, 139.6, 119.9, 88.2, 85.4, 75.8, 71.9, 62.5, 26.0, 25.8, 25.6, 18.5, 18.0, 17.8, −2.9, −4.4, −4.7, −5.0, −5.3. HRMS: *m/z* Cal. Mass for C_28_H_56_N_5_O_4_Si_3_ [M+H]^+^ = 610.3635, found [M+H]^+^ = 610.3634 (∆m= +0.0001 and error = +0.1 ppm).

**2′,3′-Bis-O-((1,1-dimethylethyl) dimethyl silyl) adenosine (6)**

Compound **5** (600 mg, 0.98 mmol) was dissolved in THF (7 mL). Then, at 0 ℃, a solution of TFA: H_2_O 1:1 (6 mL, v/v) was added, and the reaction was further stirred at the same temperature for 4h. After completion of the reaction, the mixture was quenched with aq. NaHCO_3_ (20 mL). The resulting mixture was extracted with ethyl acetate (3 × 30 mL), and the combined organic extract was dried over Na_2_SO_4_. The solvent was evaporated under reduced pressure and the crude product was purified by silica gel column chromatography (10% methanol in DCM), to obtain compound **6** as a greenish viscous solid (330 mg, 68%); R*_f_* = 0.3 (10% methanol in DCM); mp 152-154 °C; ^1^H NMR (400 MHz, CDCl_3_) δ: 8.41 (s, 1H), 8.29 (s, 1H), 5.90 (d, *J* = 6.3 Hz, 1H), 4.77-4.74 (m, 1H), 4.33 (dd, *J* = 4.4, 2.0 Hz, 1H), 4.18 (d, *J* = 1.7 Hz, 1H), 3.98 (dd, *J* = 12.8, 2.0 Hz, 1H), 3.74-3.70 (m, 1H), 0.93 (s, 9H), 0.77 (s, 9H), 0.10 (s, 6H), −0.06 (s, 3H), −0.41 (s, 3H); ^13^C NMR (100 MHz, CDCl_3_) δ: 155.3, 152.9, 149.9, 139.6, 120.0, 88.3, 85.5, 75.5, 72.8, 62.5, 26.0, 25.7, 25.6, 18.5, 18.0, 17.8, −4.4, −4.7, −5.1, −5.4; HRMS: *m/z* Cal. Mass for C_22_H_41_N_5_O_4_Si_2_ [M+H]^+^ = 496.2769, found [M+H]^+^ = 496.2769.

**2′,3′-Bis-O-((1,1-dimethylethyl) dimethyl silyl)-5′-O-sulfamoyl adenosine (7)** [4]

Formic acid (0.1 mL, 2.47 mmol), was added to ice-cold chlorosulfonyl isocyanate (350 mg, 2.47 mmol) and allowed to stir at 0 ℃ for 5 min. The resulting solid was dissolved in acetonitrile (2 mL) and added to the solution of compound **6** (500 mg, 1.01 mmol) in dimethyl acetamide (5 mL) at 0 ℃. Then the reaction mixture was further stirred at rt for 1 h, and the reaction was quenched by the addition of triethylamine (1.5 mL) and methanol (2 mL) in a sequential way with 5-10 min of wait time. Volatiles were evaporated under reduced pressure, and the crude product was diluted ethyl acetate. Then, the crude solution was extracted from aq. NaHCO_3_ (30 mL) in ethyl acetate (2 x 15 mL), and the combined organic extract was dried over Na_2_SO_4_. The solvent was evaporated under reduced pressure, and the crude compound was purified by silica gel column chromatography (60% ethyl acetate in pet ether) to obtain compound **7** as a white solid (377 mg, 65%); R*_f_* = 0.3 (60% ethyl acetate in pet ether); mp 161-164 °C; ^1^H NMR (500 MHz, CDCl_3_) δ: ^1^H NMR (400 MHz, CDCl_3_) δ: 8.35(s, 1H), 8.15 (s, 1H), 7.65 (s, 2H), 7.31 (s, 2H), 5.95 (d, *J* = 6.5 Hz, 1H), 4.91-4.94 (m, 1H), 4.44-4.28 (m, 2H), 4.19-4.16 (m, 1H), 3.38 (s, 1H), 0.90 (s, 9H), 0.70 (s, 9H), 0.14 (s, 3H), 0.12 (s, 3H), −0.08 (s, 3H), −0.37 (s, 3H); ^13^C NMR (100 MHz, CDCl_3_) δ: 156.5, 153.2, 149.4, 140.2, 119.7, 87.7, 83.6, 74.2, 72.8, 68.5, 26.1, 25.9, 18.2, 17.9, −4.2, −4.3, −4.4, −5.1; HRMS: *m/z* Cal. Mass for C_22_H_43_N_6_O_6_SSi_2_ [M+H]^+^ = 575.2498, found [M+H]^+^ = 575.2497 (∆m= +0.0001 and error = +0.1 ppm).

**4-(*tert-*Butyl) 1-(2,3,5,6-tetraflurophenyl) (((9*H*-fluoren-9-yl) methoxycarbonyl)-l-aspartate (8)**

Commercially available Fmoc-L-Asp(O*t*Bu)-OH (500 mg, 1.21 mmol) was dissolved in DMF (5 mL), followed by the addition of tetraflurophenol (302 mg, 1.82 mmol) and EDC.HCl (465 mg. 2.43 mmol). The reaction was stirred at rt for 16 h. After consumption of the starting material, the reaction was quenched with cold, aq. NaHCO_3_ (30 mL). The product was extracted in ethyl acetate (3 × 20 mL), the combined organic extract was dried over Na_2_SO_4_, and the solvent was removed under reduced pressure. The crude product was purified by silica gel column chromatography (10% ethyl acetate in pet ether), to obtain compound **8** as white solid (380 mg, 67%); R*_f_* = 0.3 (15% ethyl acetate in pet ether); mp: 93-95 ℃; ^1^H NMR (400 MHz, CDCl_3_) δ: 7.76 (d, *J* = 7.8 Hz, 2H), 7.60 (dd, *J* = 7.3, 6.2 Hz, 2H), 7.39 (t, *J* = 7.5 Hz, 2H) 7.32-7.28 (m, 2H), 7.06-6.98 (m, 1H), 5.02-4.98 (m, 1H), 4.51-4.22 (m, 3H), 3.16 (dd, *J* = 17.5, 5.1 Hz, 1H), 2.91 (dd, *J* = 17.5, 4.4 Hz, 1H), 1.49 (s, 9H); ^13^C NMR (100 MHz, CDCl_3_) δ: 169.8, 167.3, 156.2, 143.6, 143.5, 141.3, 127.8, 127.2, 127.1, 125.1, 125.07, 120.0, 103.9, 103.7, 103.4, 96.1, 95.9, 95.6, 82.8, 67.7, 50.3, 47.0, 37.6, 27.9; HRMS: *m/z* Cal. Mass for C_29_H_25_NO_6_F_4_ [M+H]^+^ = 561.1614, found [M+H]^+^ = 561.1614

**5′-O-[*N*-(L-Aspartate)-sulfamoyl] adenosine (Asp-AMS)**

Compound **7** (130 mg, 0.22 mmol) was dissolved in DMF (2 mL) followed by the addition of DIPEA (0.5 mL, 2.89 mmol). The solution was stirred for 2 min, and then activated tetraflurophenyl-aspartate **8** (163 mg, 0.29 mmol) was added. The reaction mixture was stirred at rt for 24 h. After consumption of the starting material, piperidine (0.3 mL) was added to the reaction mixture and stirred for 45 min. The volatiles were evaporated, and the crude compound **9** was dissolved in THF, then treated with TBAF (0.1 M in THF) (0.1 mL, 0.44 mmol), and the progress of the reaction was monitored by TLC. After the deprotection of TBS groups, the volatiles were evaporated, and the crude reaction mixture dissolved in DCM (5 mL). Then 4N HCl in dioxane (0.8 mL, v/v) was added, and the reaction mixture was further stirred at rt for 3 h. The solvent was evaporated, and the crude product was subjected to RP-HPLC purification. A linear gradient from 2-20% acetonitrile in 60 min. at a flow rate of 5 mL/minute to get the compound, which was lyophilized to obtain **Asp-AMS** as a white solid. (22 mg, 21%). ^1^H NMR (400 MHz, DMSO-*d*_6_) δ: 8.40 (s, 1H), 8.15 (s, 1H), 7.29 (s, 2H), 5.9 (d, *J* = 5.42 Hz, 1H), 4.58 (d, *J* = 6.2 Hz, 1H), 4.15 (t, *J* = 3.2 Hz, 1H), 4.10-4.01 (m, 4H), 2.34-3.27 (m, 2H); ^13^C NMR (100 MHz, DMSO-*d*_6_) δ: 173.5, 173.1, 156.4, 153.1, 150.0, 139.8, 119.3, 87.4, 82.9, 74.0, 71.0, 68.0, 53.2, 36.7; HRMS: *m/z* Cal. Mass for C_14_H_19_N_7_O_9_S [M+H]^+^ = 462.1038, found [M+H]^+^ = 462.1037 (∆m= +0.0001 and error = +0.2 ppm).

**^1^H NMR (800 MHz, methanol-*d*_4_) of compound 3**

**
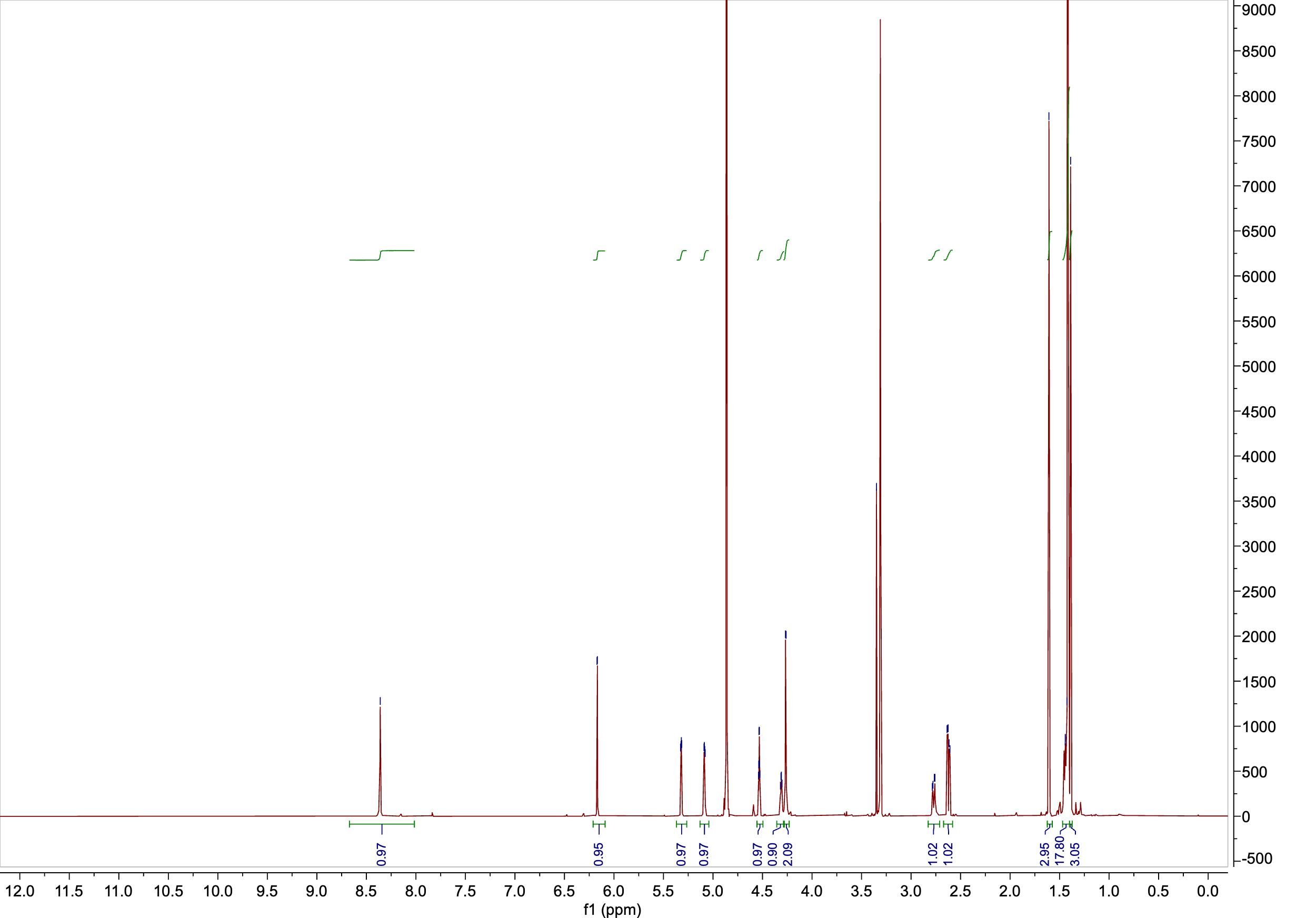
**

**^13^C{H} NMR (200 MHz, methanol-*d*_4_) of compound 3**
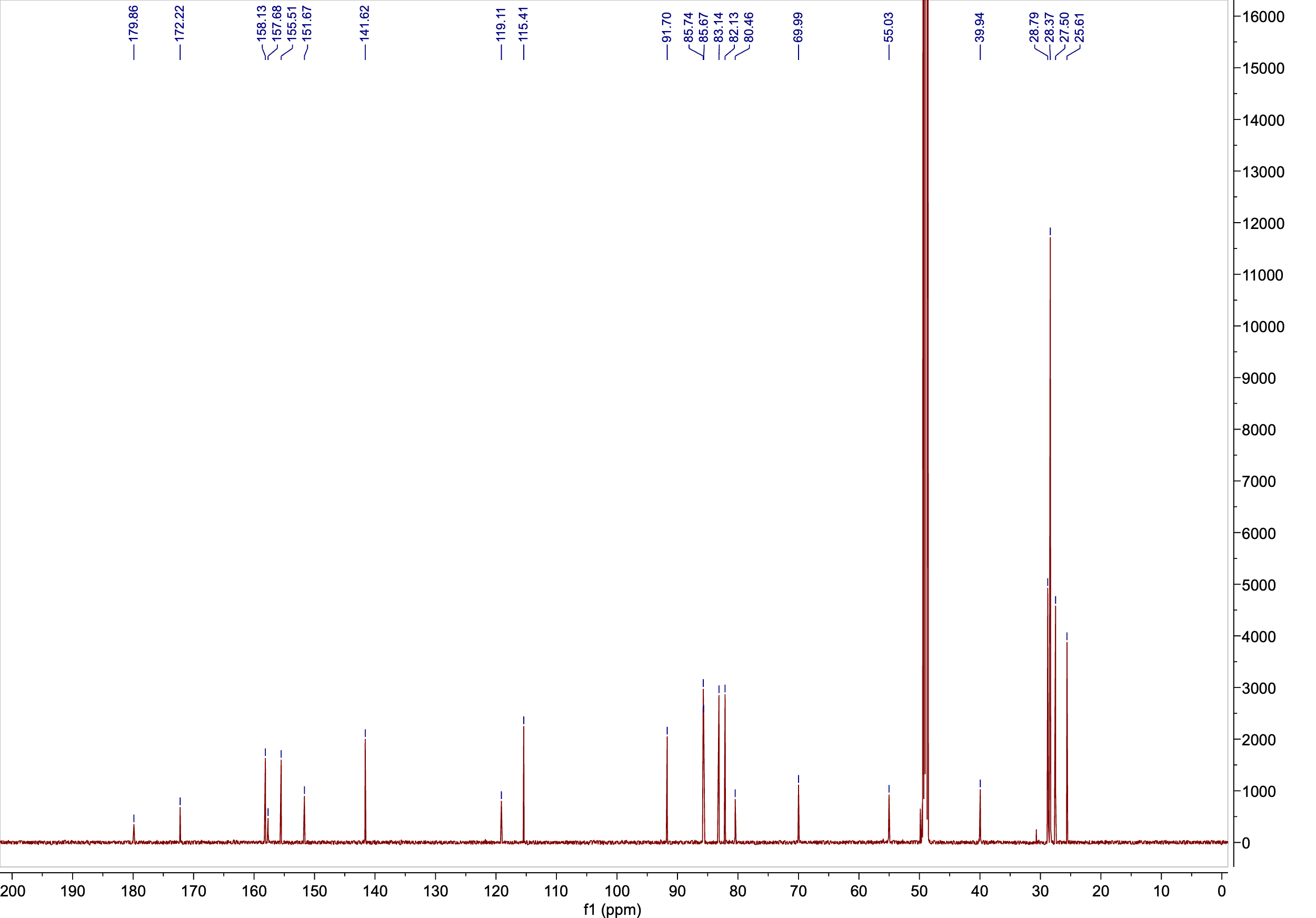


**^1^H NMR (500 MHz, DMSO-*d*_6_) of Asp-DACM**


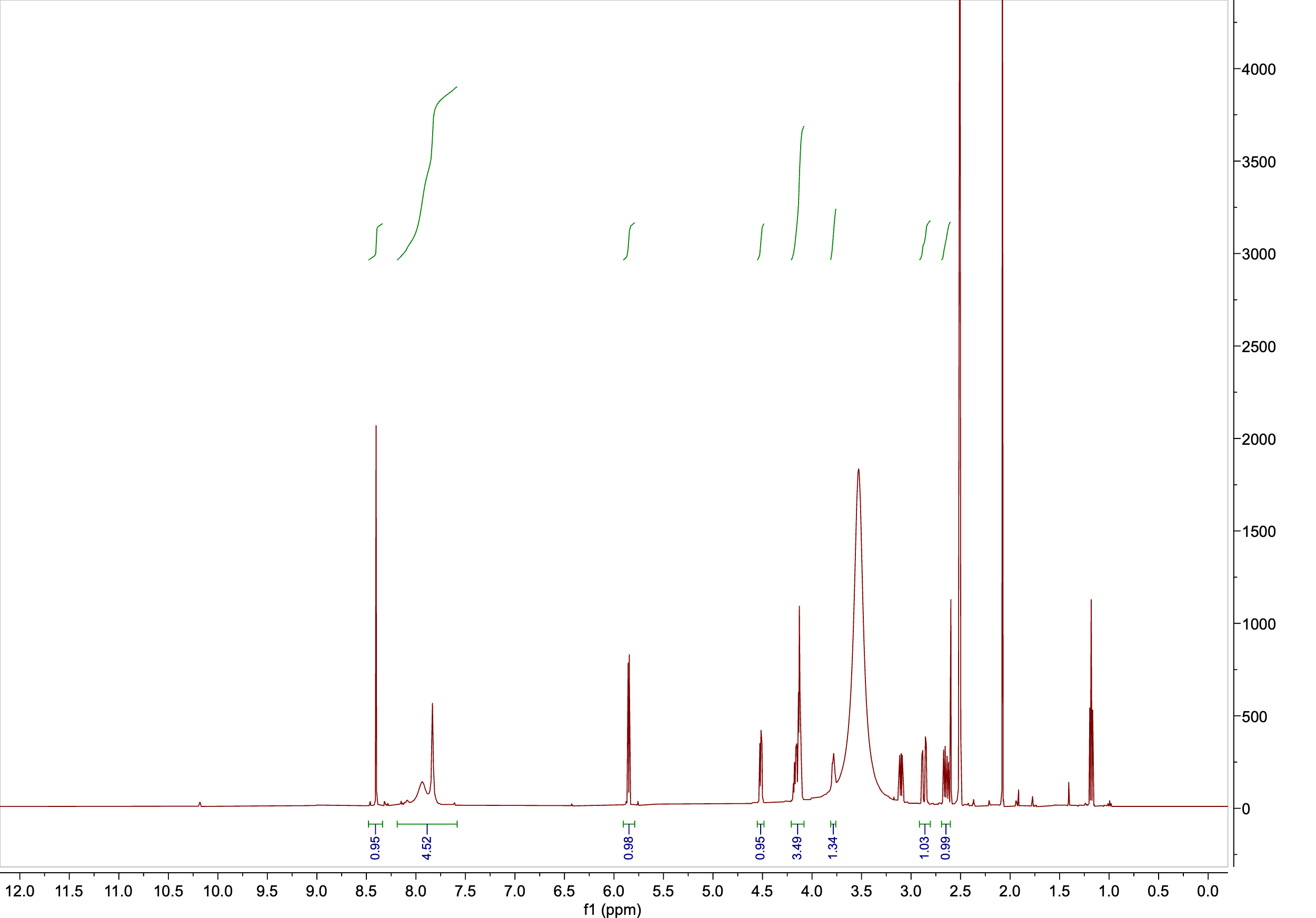


**^13^C NMR (500 MHz, DMSO-*d*_6_) of Asp-DACM**

**
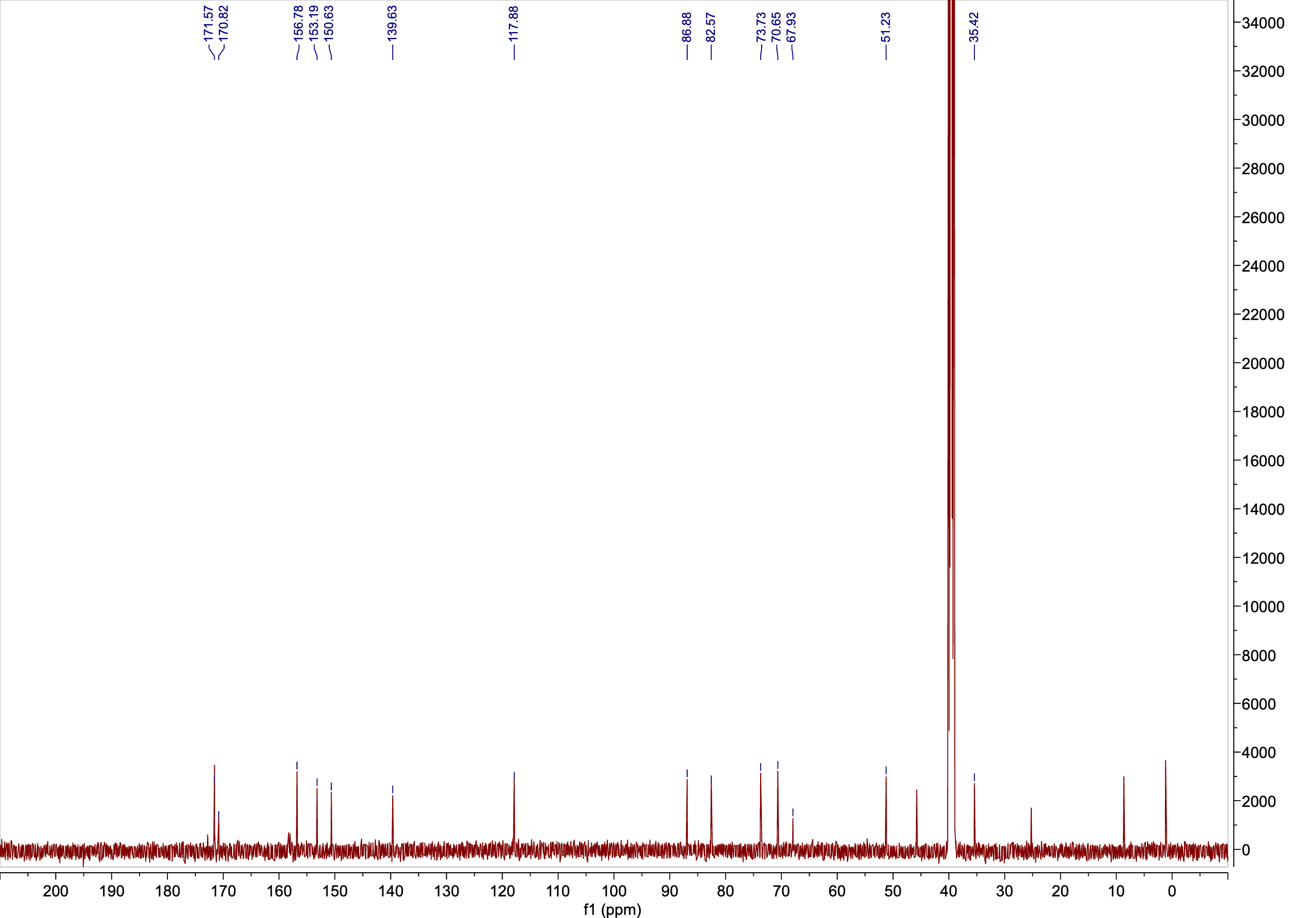
**

**^1^H NMR (400 MHz, CDCl_3_) of 2′,3′,5′-tris-O-((1,1-dimethylethyl) dimethyl silyl) adenosine (5)**

**
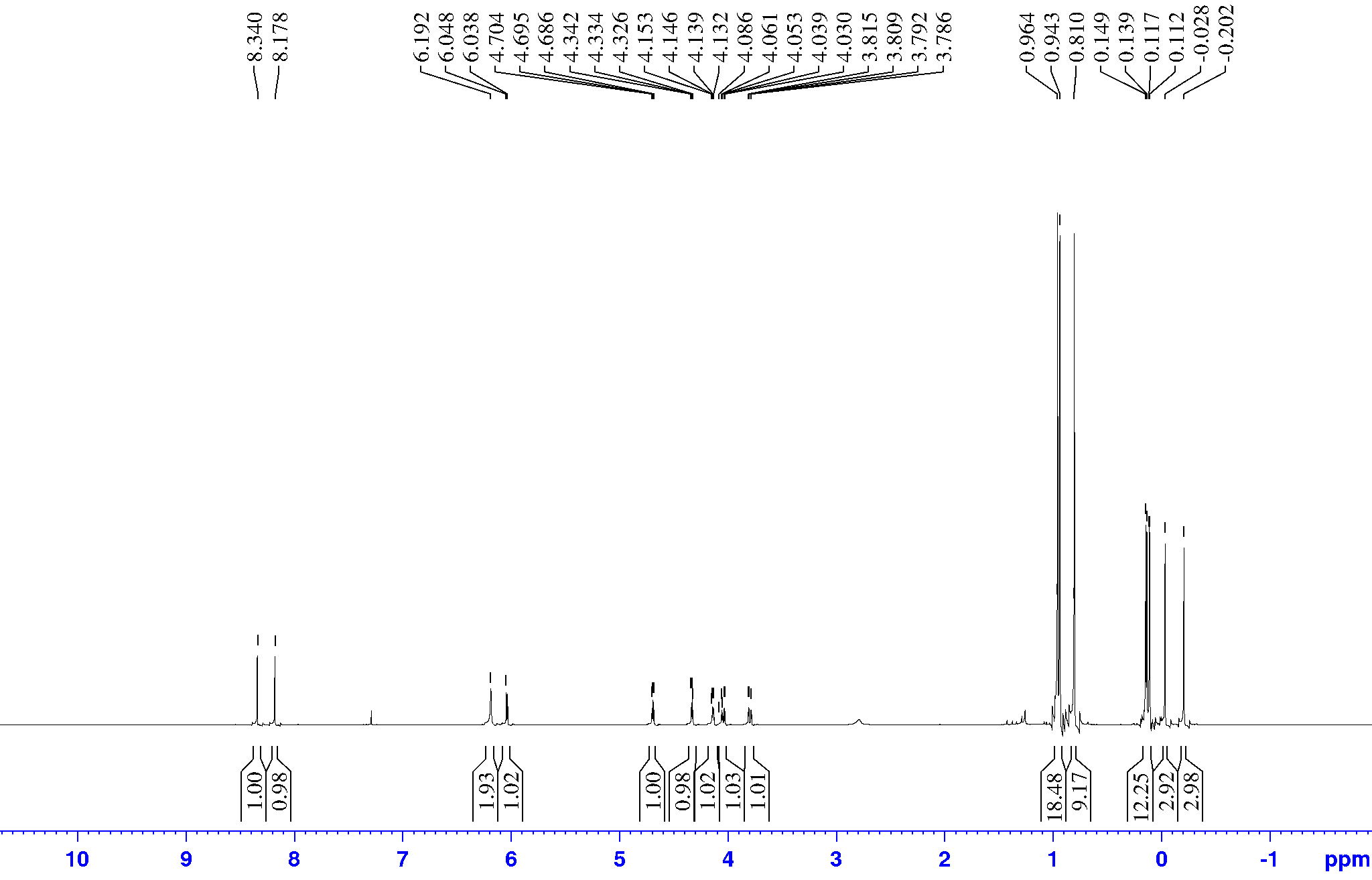
**

**^13^C{^1^H} NMR (100 MHz, CDCl_3_) of 2′,3′,5′-tris-O-((1,1-dimethylethyl) dimethyl silyl) adenosine (5)**


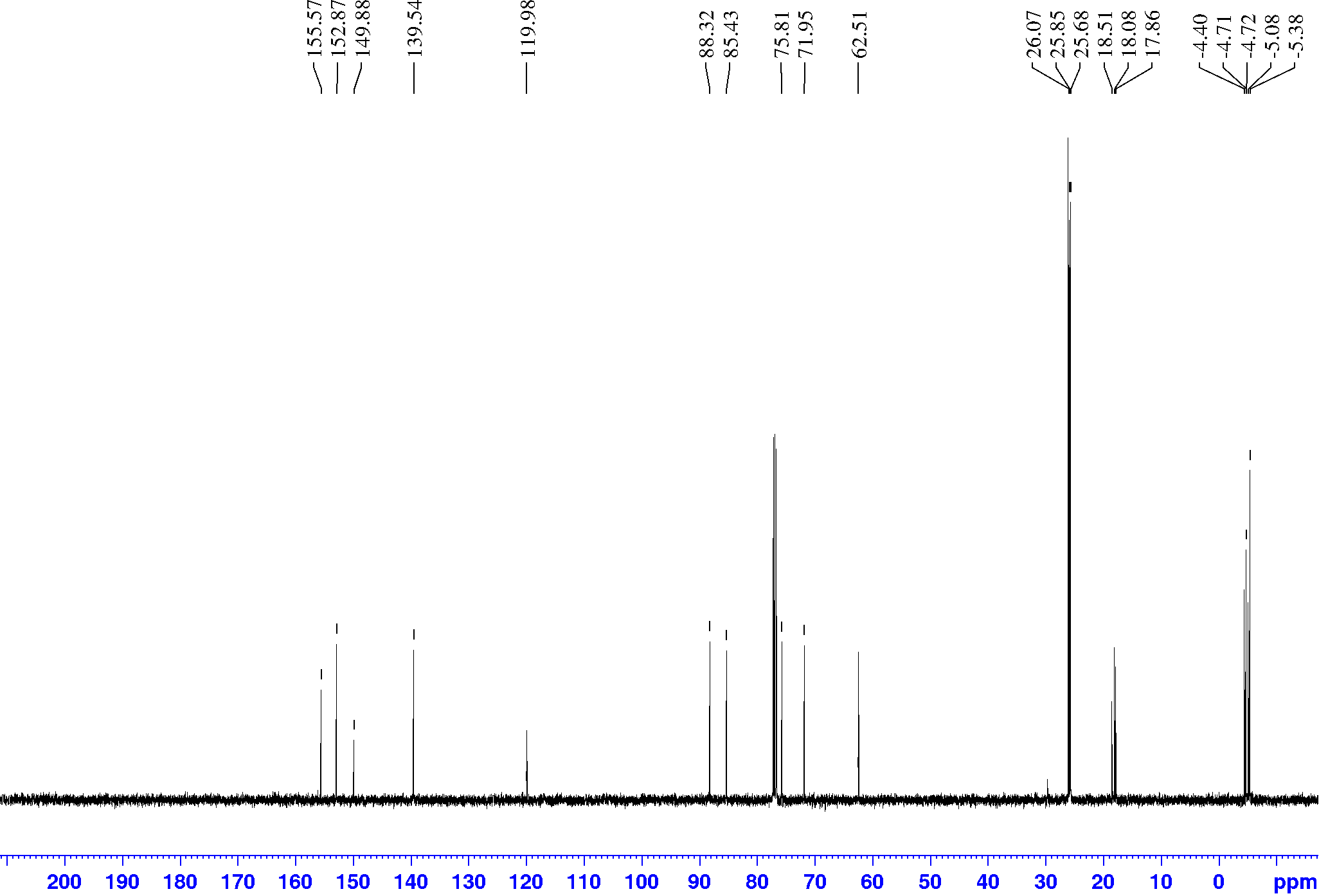

**^1^H NMR (400 MHz, CDCl_3_) of 2′,3′-bis-O-((1,1-dimethylethyl) dimethyl silyl) adenosine (6)**

**
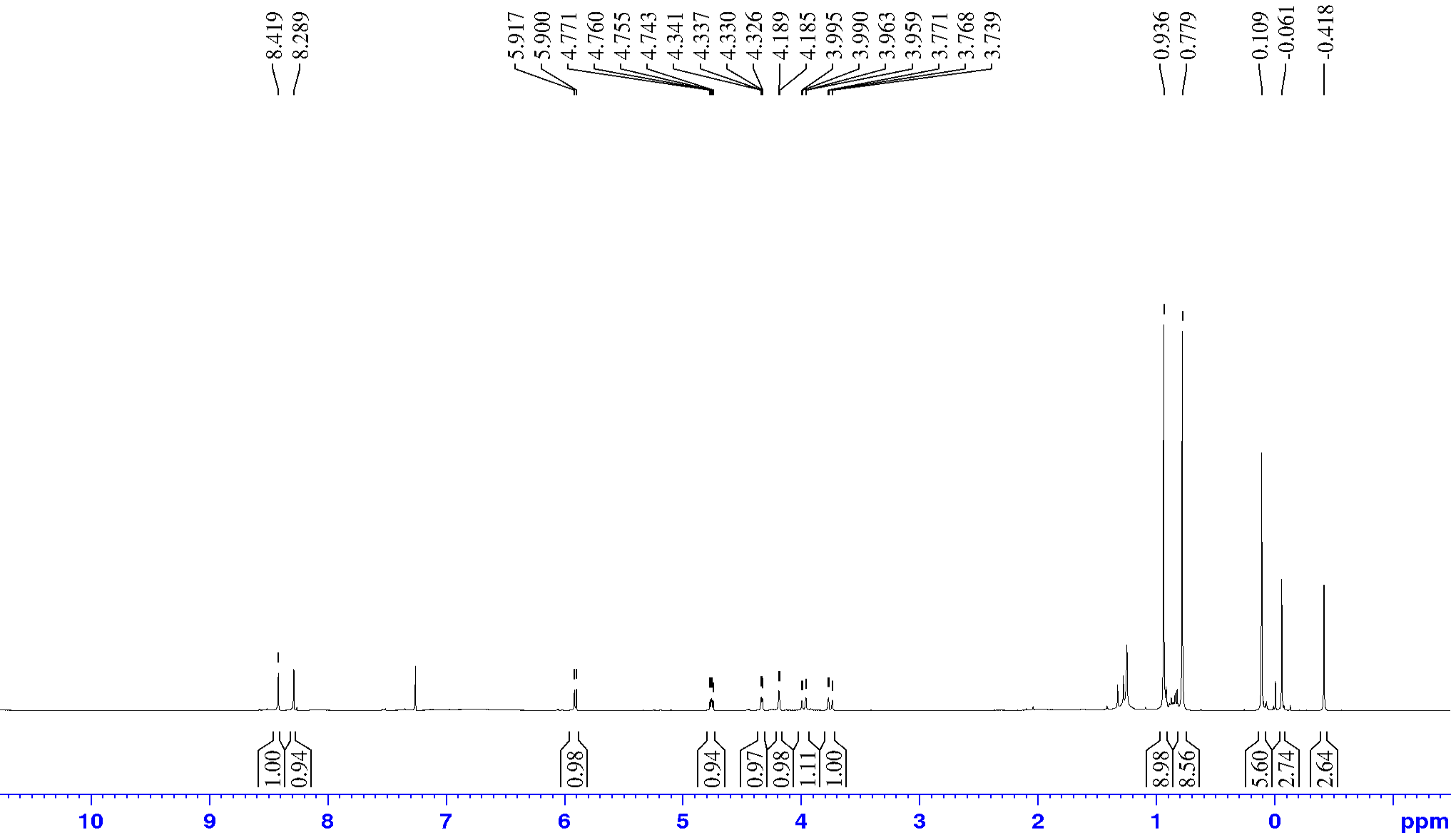
**

**^13^C{^1^H} NMR (100 MHz, CDCl_3_) of 2′,3′-bis-O-((1,1-dimethylethyl) dimethyl silyl) adenosine (6)**


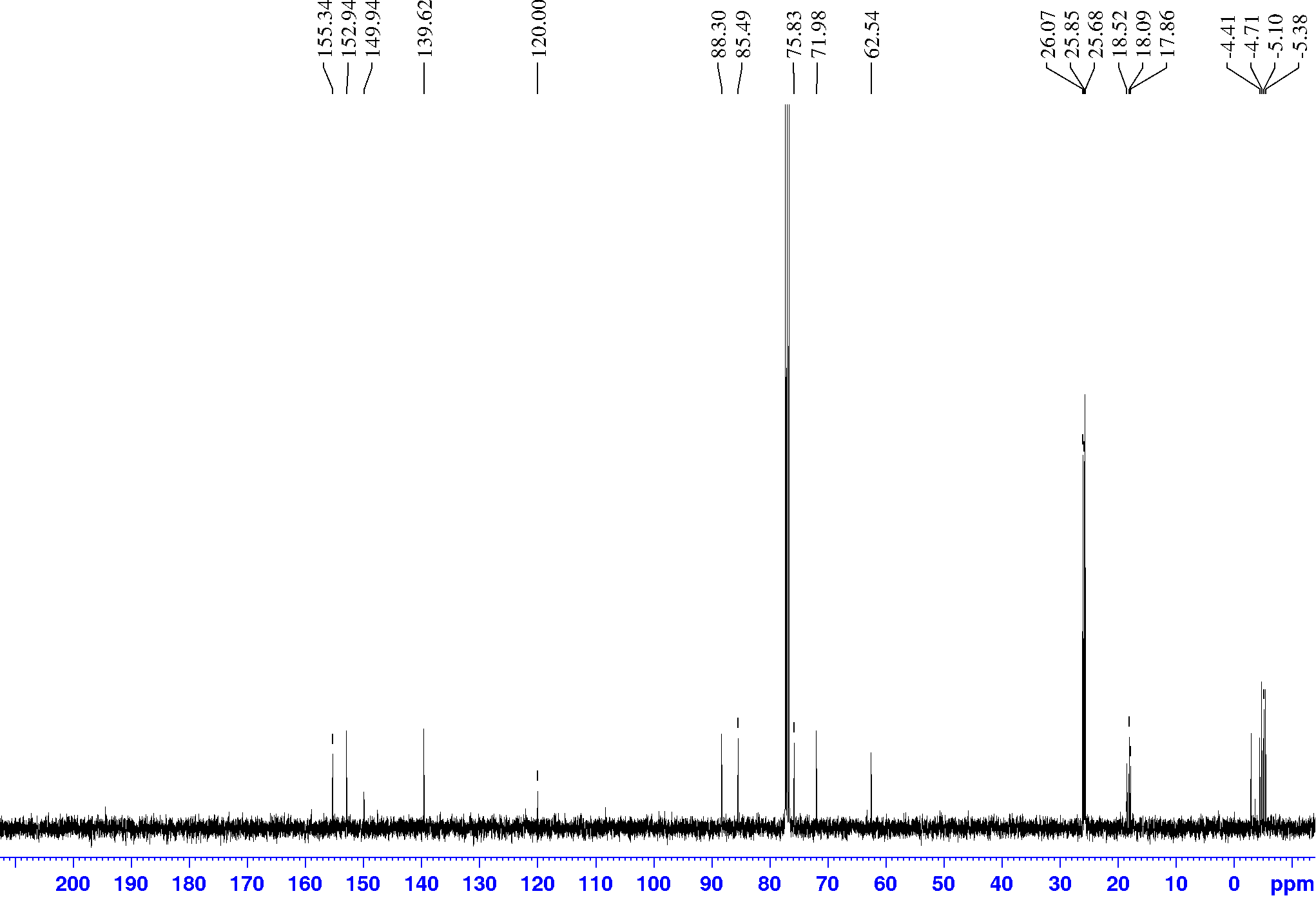

**^1^H NMR (400 MHz, DMSO-*d*_6_) of 2′,3′-bis-O-((1,1-dimethylethyl) dimethyl silyl)-5′-O-sulfamoyl adenosine (7)**


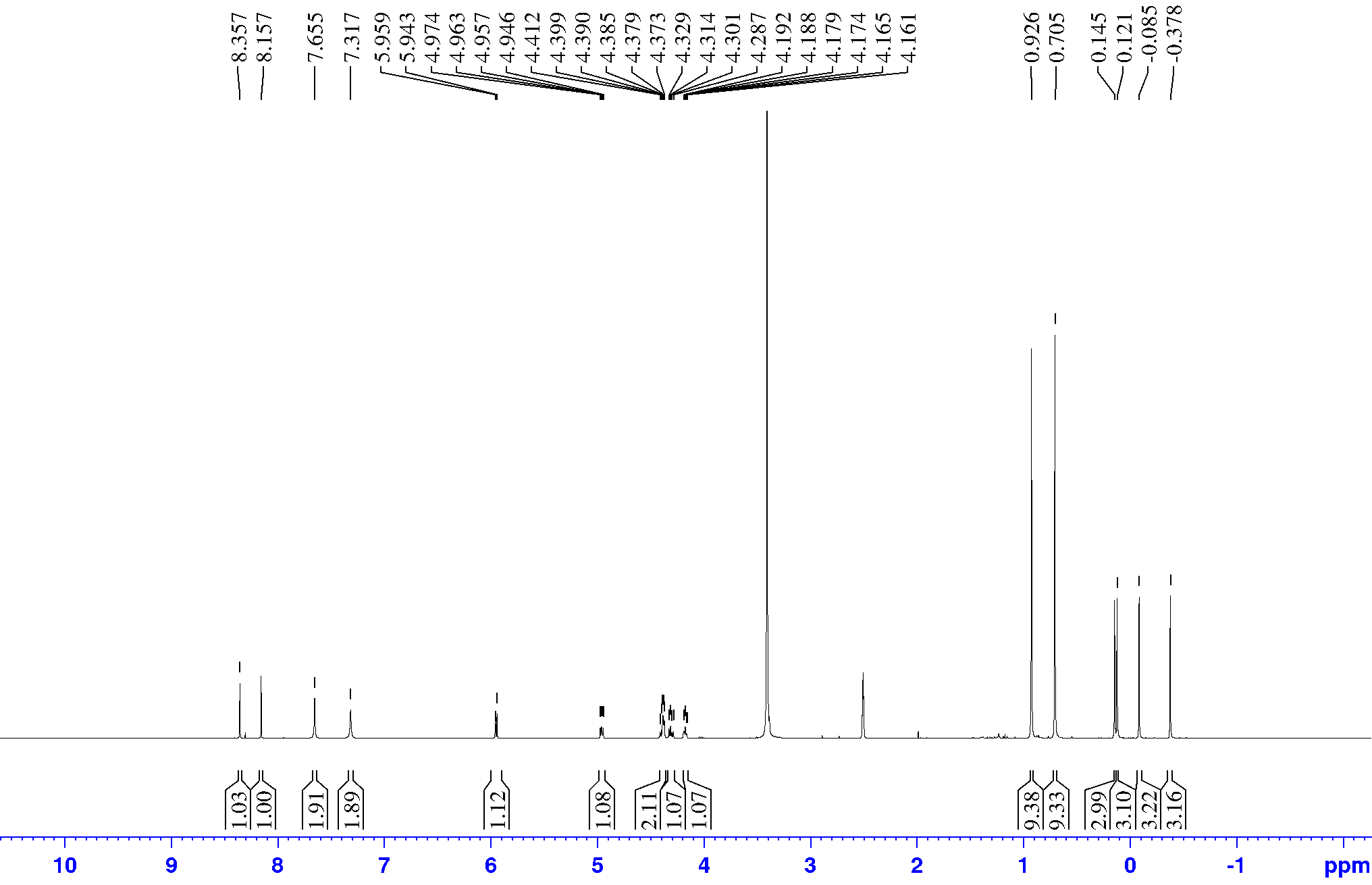

H_2_O

**^13^C{^1^H} NMR (100 MHz, DMSO-*d*_6_) of 2′,3′-bis-O-((1,1-dimethylethyl) dimethyl silyl)-5′-O-sulfamoyl adenosine (7)**


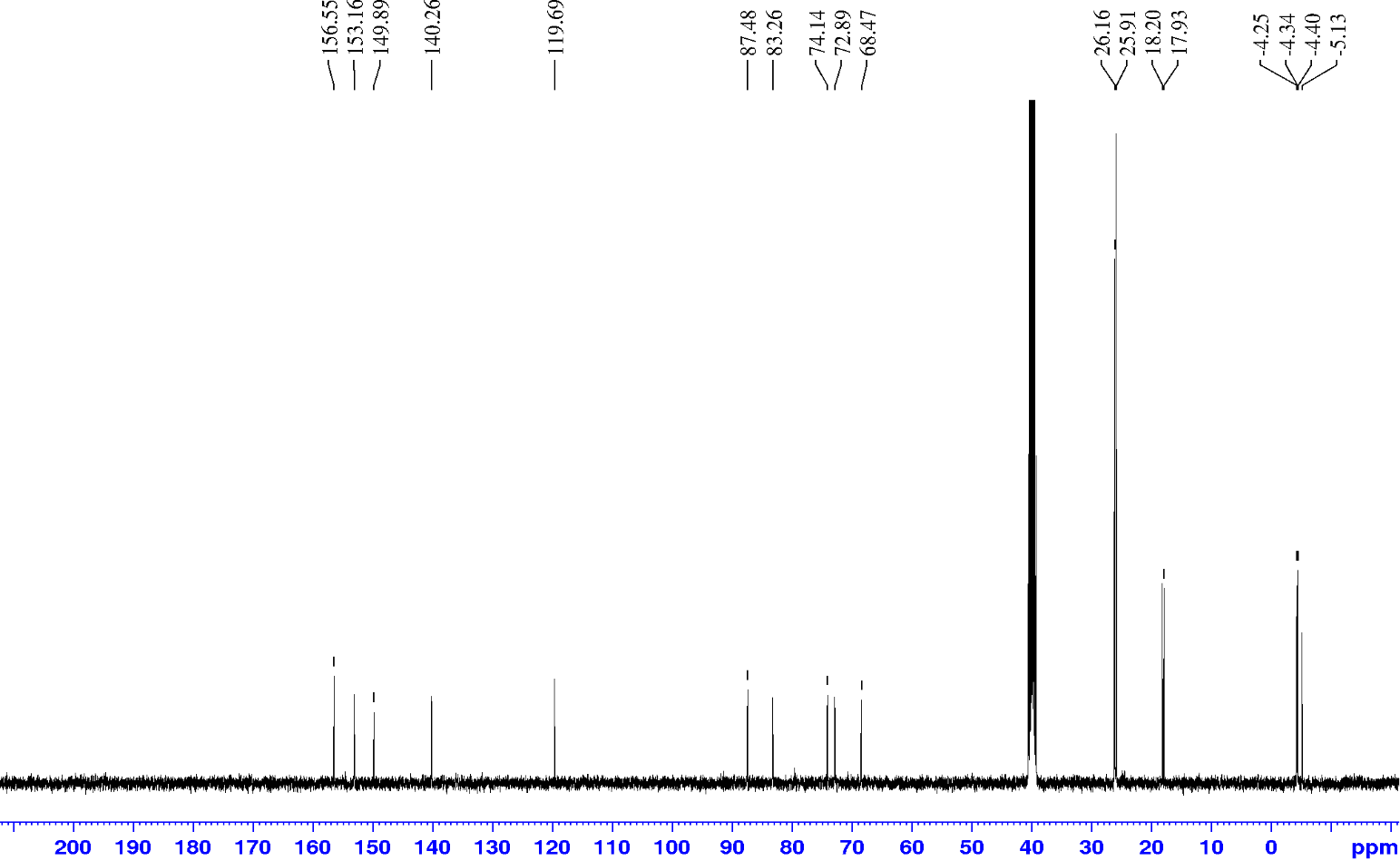

**^1^H NMR (400 MHz, CDCl_3_) of 4-(tert-butyl) 1-(2,3,5,6-tetraflurophenyl) (((9*H*-fluoren-9-yl) methoxycarbonyl)-L-aspartate (8)**

**
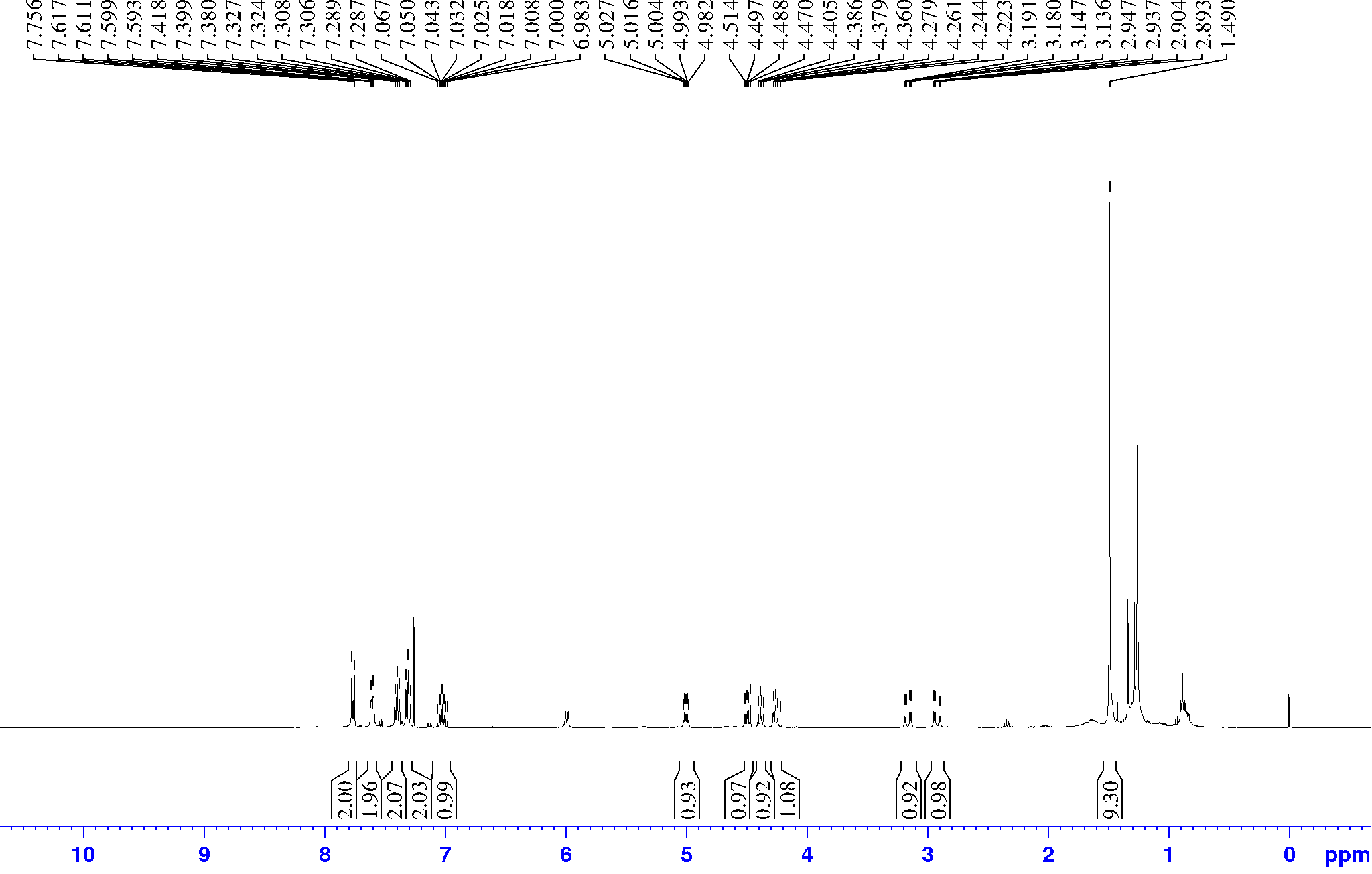
**

**^13^C{^1^H} NMR (100 MHz, CDCl_3_) of 4-(tert-butyl) 1-(2,3,5,6-tetraflurophenyl) (((9*H*-fluoren-9-yl) methoxycarbonyl)-L-aspartate (8)**

**
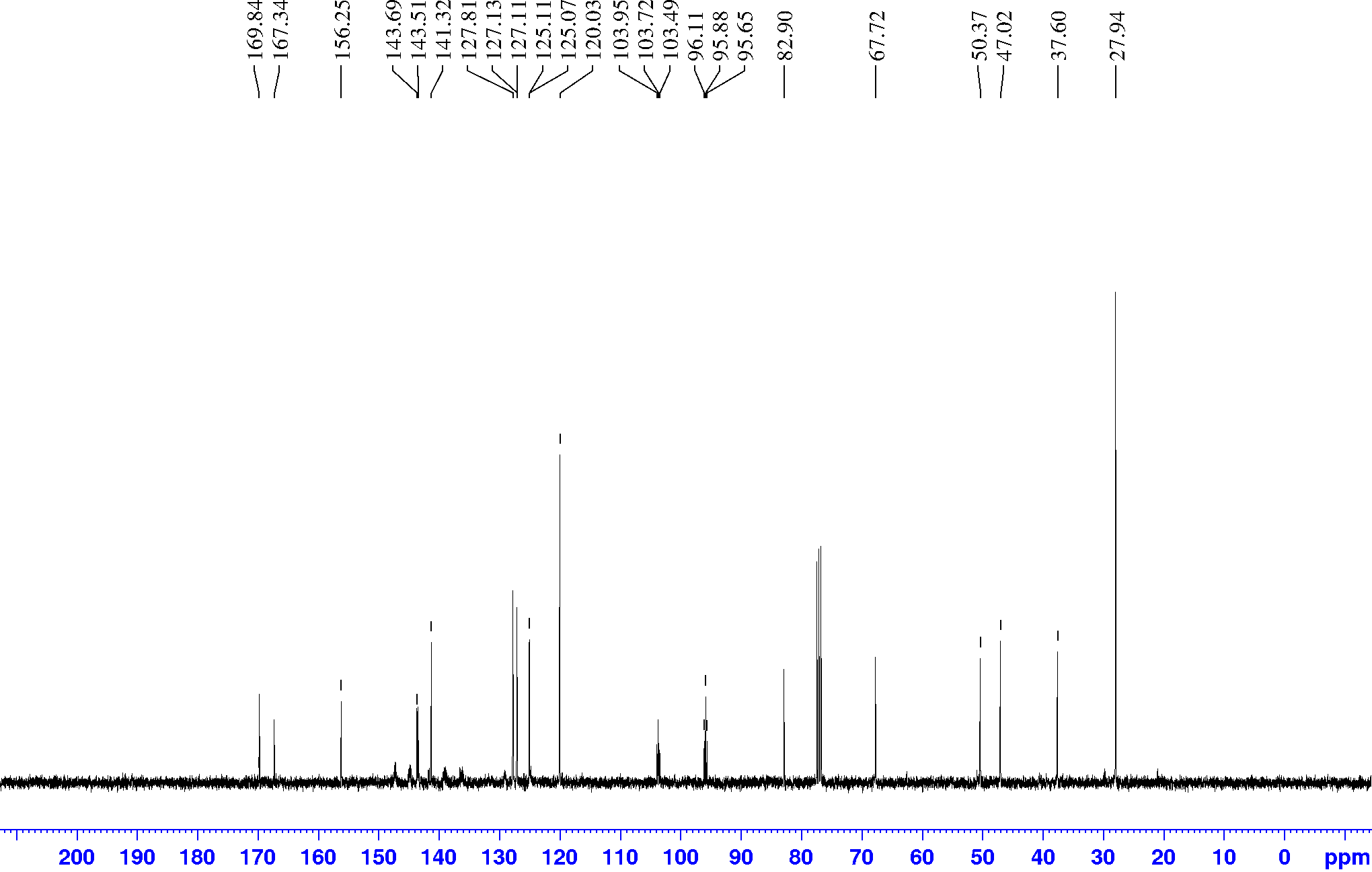
**

**^1^H NMR (400 MHz, DMSO-*d*_6_) of 5′-O-[*N*-(L-Aspartate)-sulfamoyl] adenosine (Asp-AMS)**

**
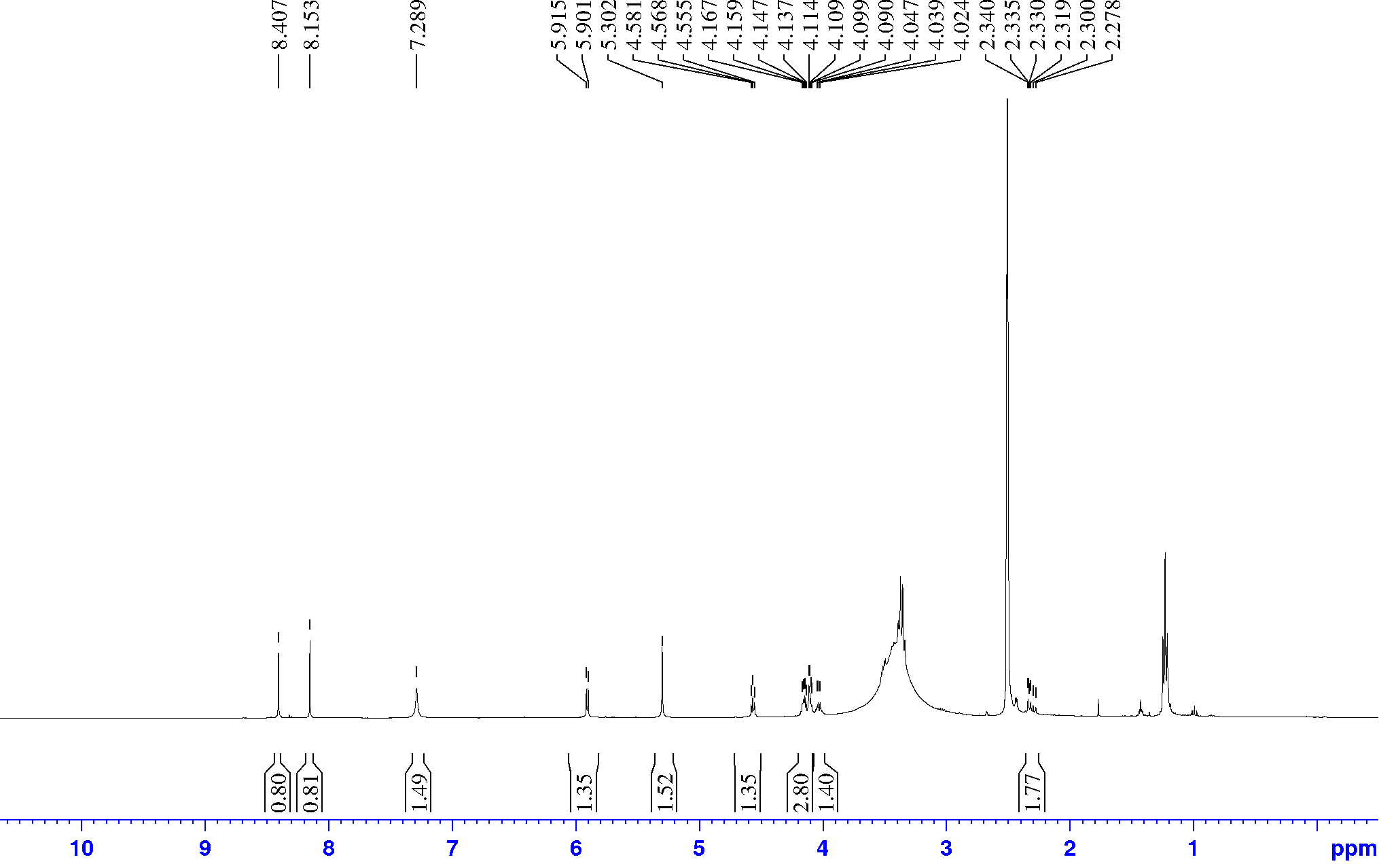
**

**^13^C{^1^H} NMR (100 MHz, DMSO-*d*_6_) of 5′-O-[*N*-(L-Aspartate)-sulfamoyl] adenosine (Asp-AMS)**


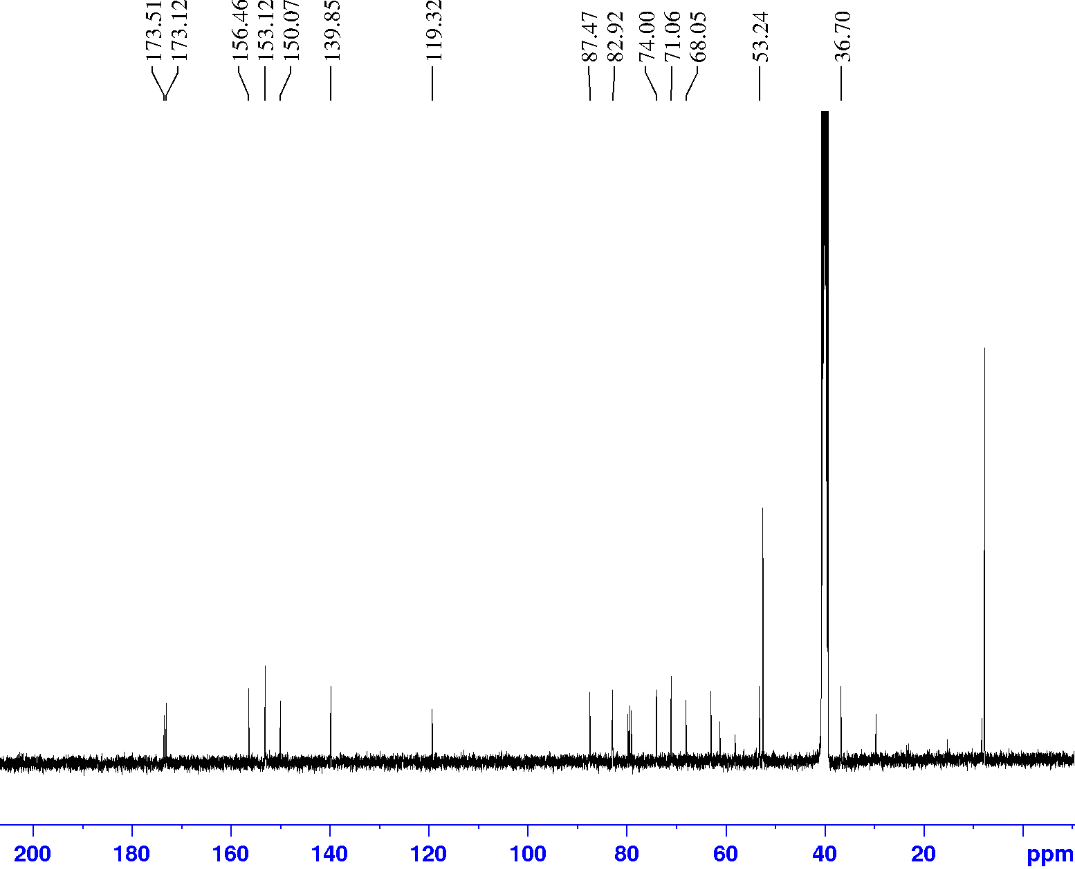

I

HPLC chromatogram of purified **Asp-AMS**. Column and conditions Shim-pack GIST C18 column (5 µM, 250 mm length, 4.6 mm I.D); Mobile phase A: water, mobile phase B: acetonitrile. Gradient: 2-20% B in 60 min. Flow rate 1 mL/min. HPLC chromatogram shows the major peak **Asp-AMS** at a retention time of 22.5 min.

| 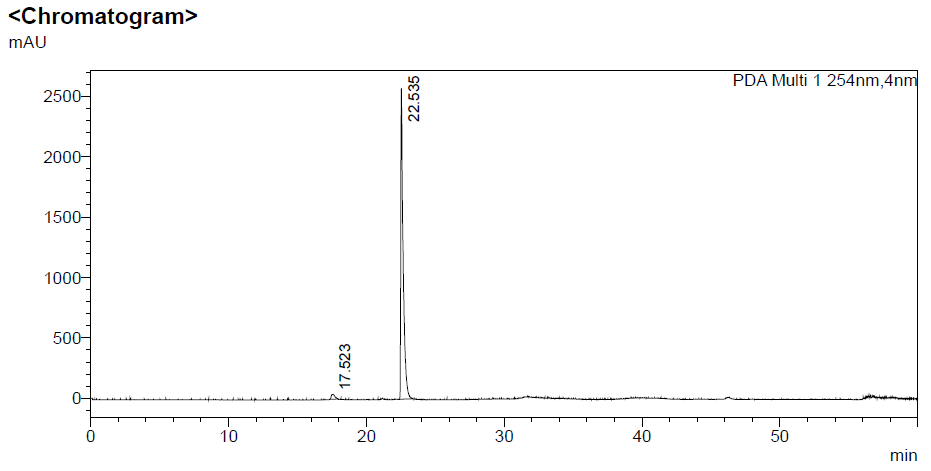 |
| --- |

**References**

1. Cain R, Salimraj R, Punekar AS, Bellini D, Fishwick CWG, Czaplewski L, et al. Structure-guided enhancement of selectivity of chemical robe inhibitors targeting bacterial seryl-tRNA synthetase. J Med Chem. 2019;62(21):9703-17

2. Mujumdar P, Bua S, Supuran CT, Peat TS, Poulsen SA. Synthesis, structure and bioactivity of primary sulfamate-containing natural products. Bioorg Med Chem Lett. 2018;28(17):3009-13

3. Voorneveld J, Rack JGM, van Gijlswijk L, Meeuwenoord NJ, Liu Q, Overkleeft HS, et al. Molecular tools for the study of ADP-ribosylation: a unified and versatile method to synthesise native mono-ADP-ribosylated peptides. Chemistry. 2021;27(41):10621-7

4. Gadakh B, Vondenhoff G, Lescrinier E, Rozenski J, Froeyen M, Van Aerschot A. Base substituted 5'-O-(N-isoleucyl)sulfamoyl nucleoside analogues as potential antibacterial agents. Bioorg Med Chem. 2014;22(10):2875-86
